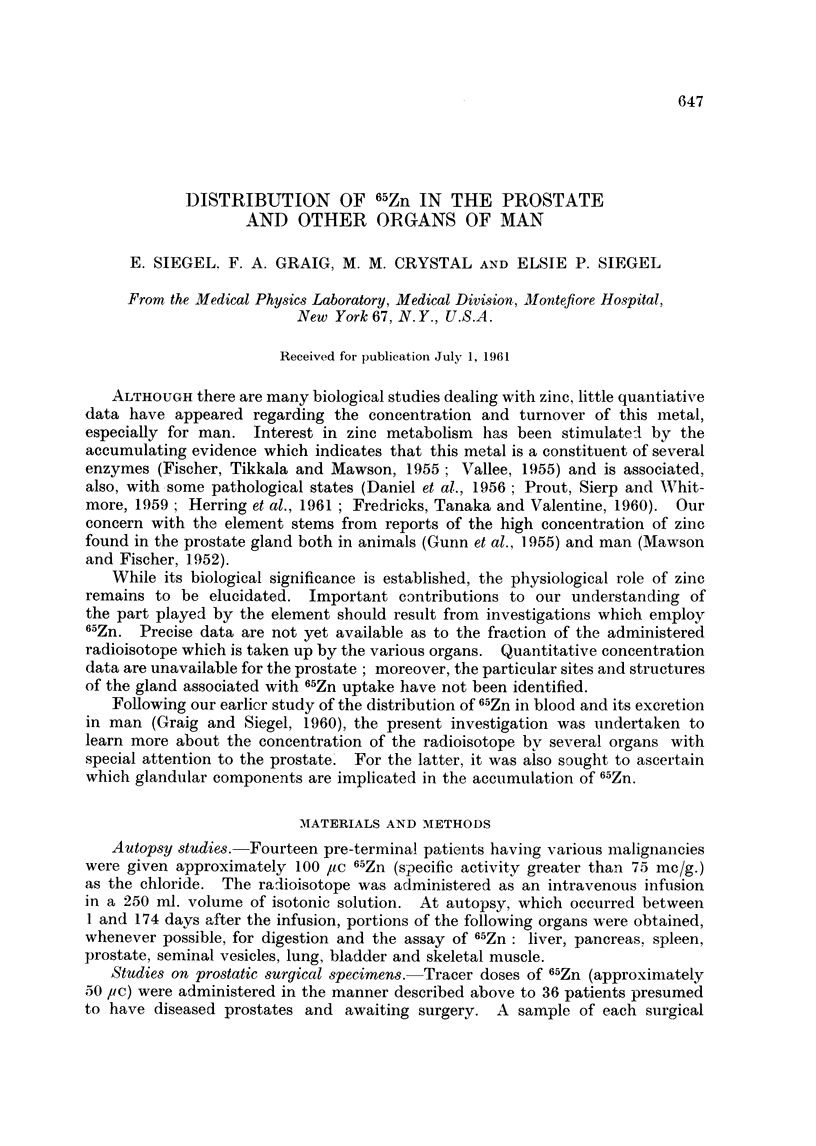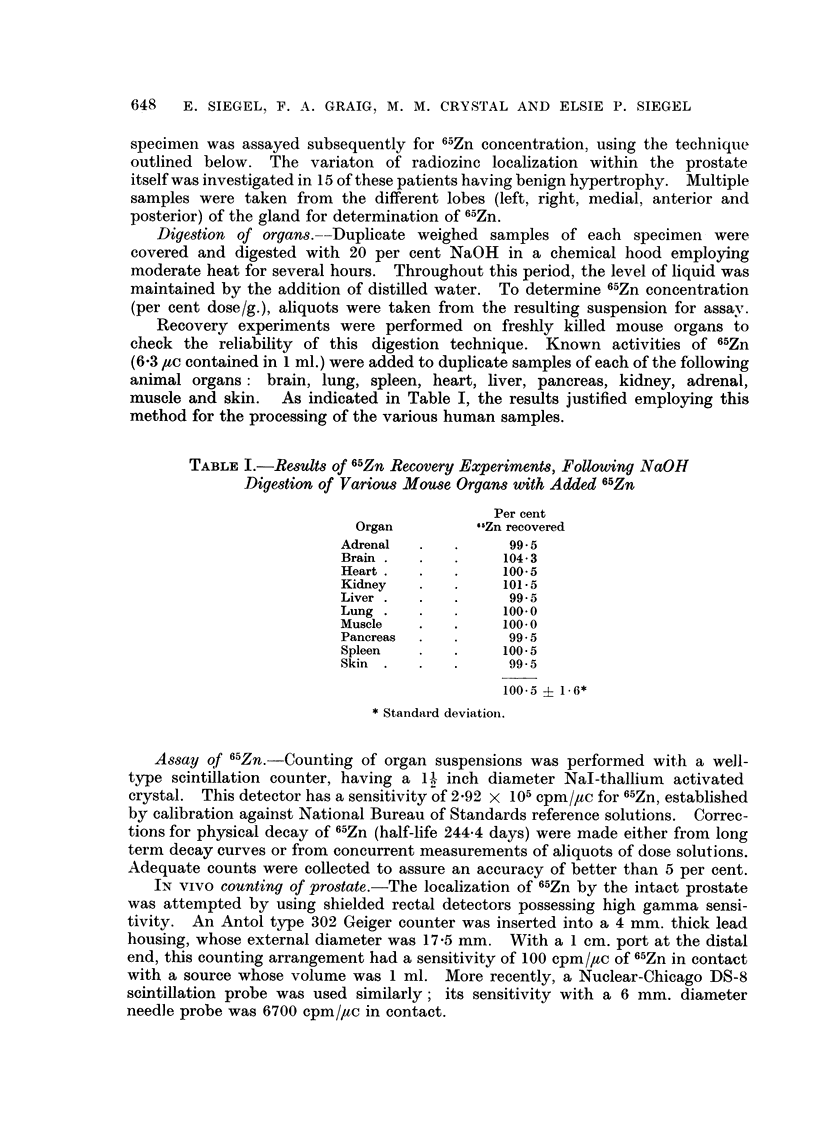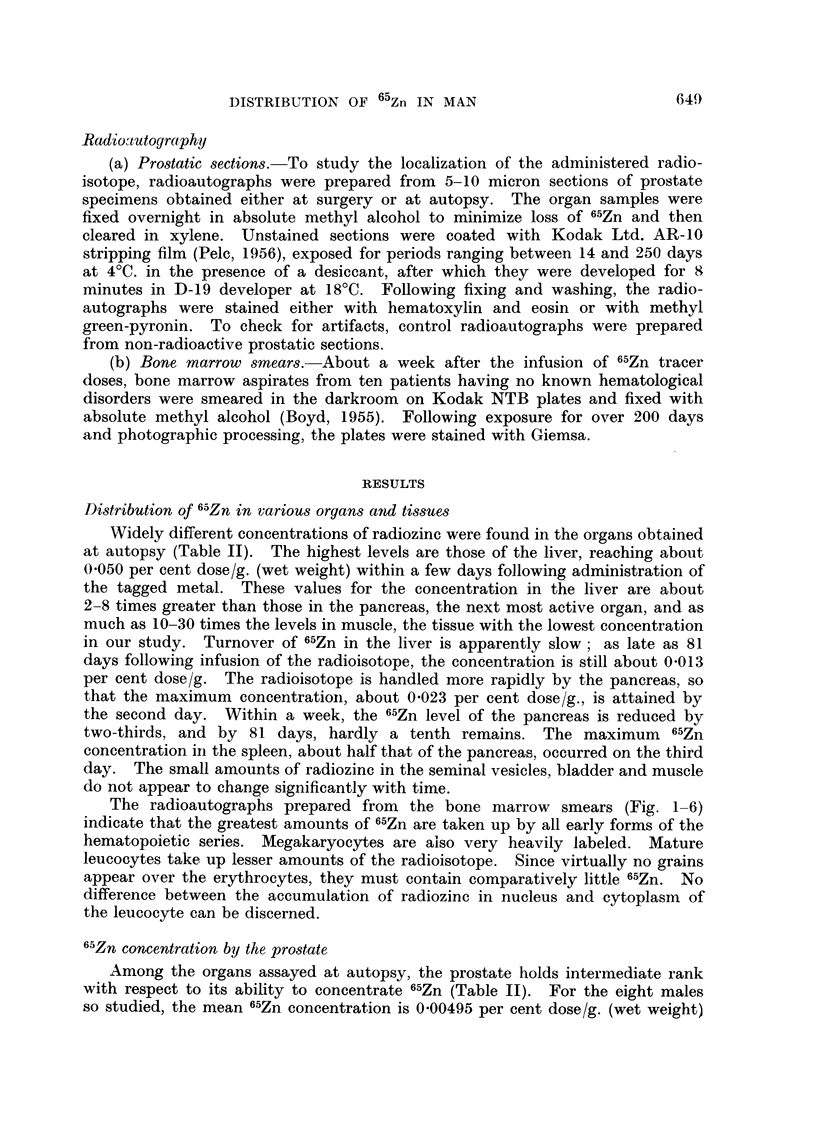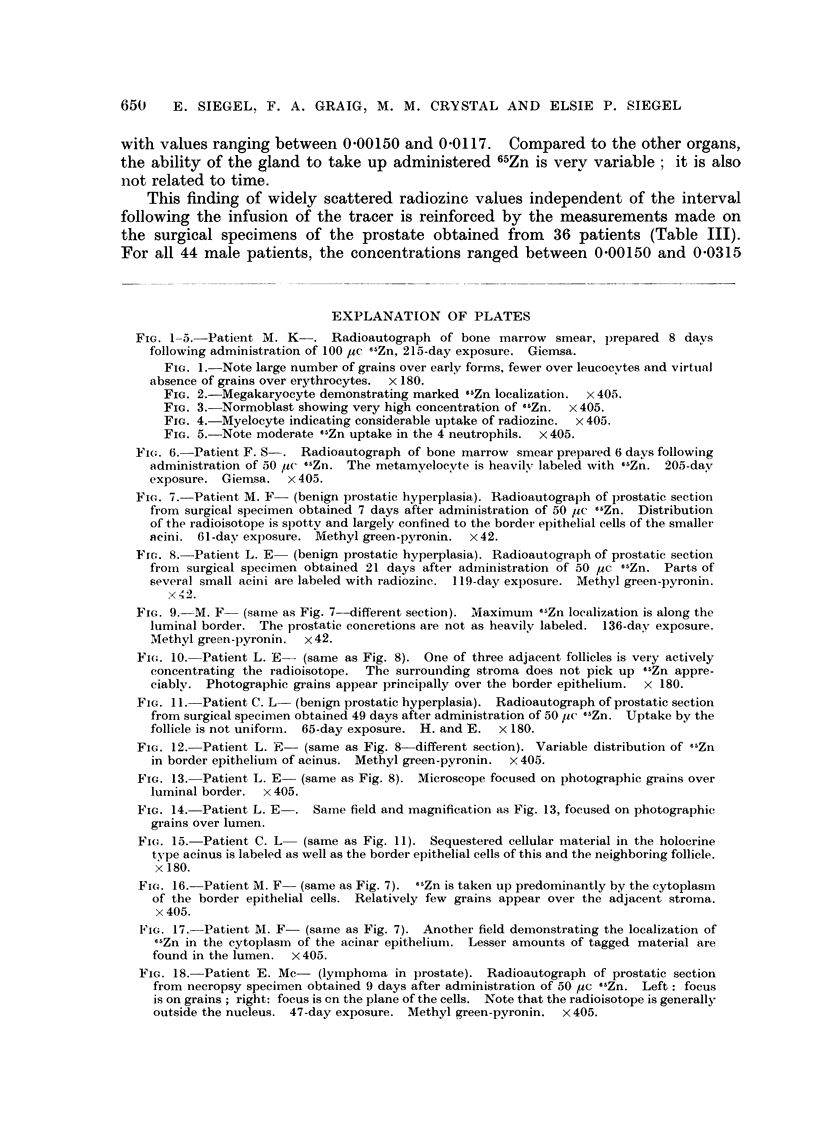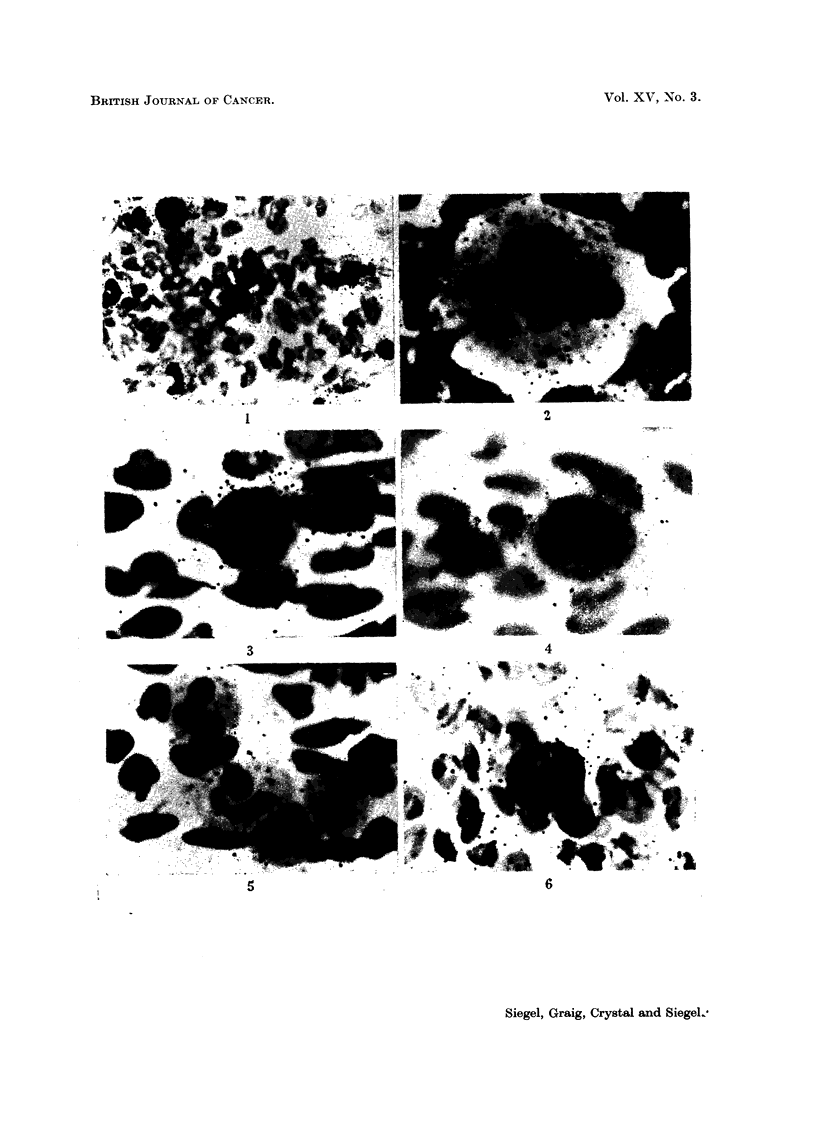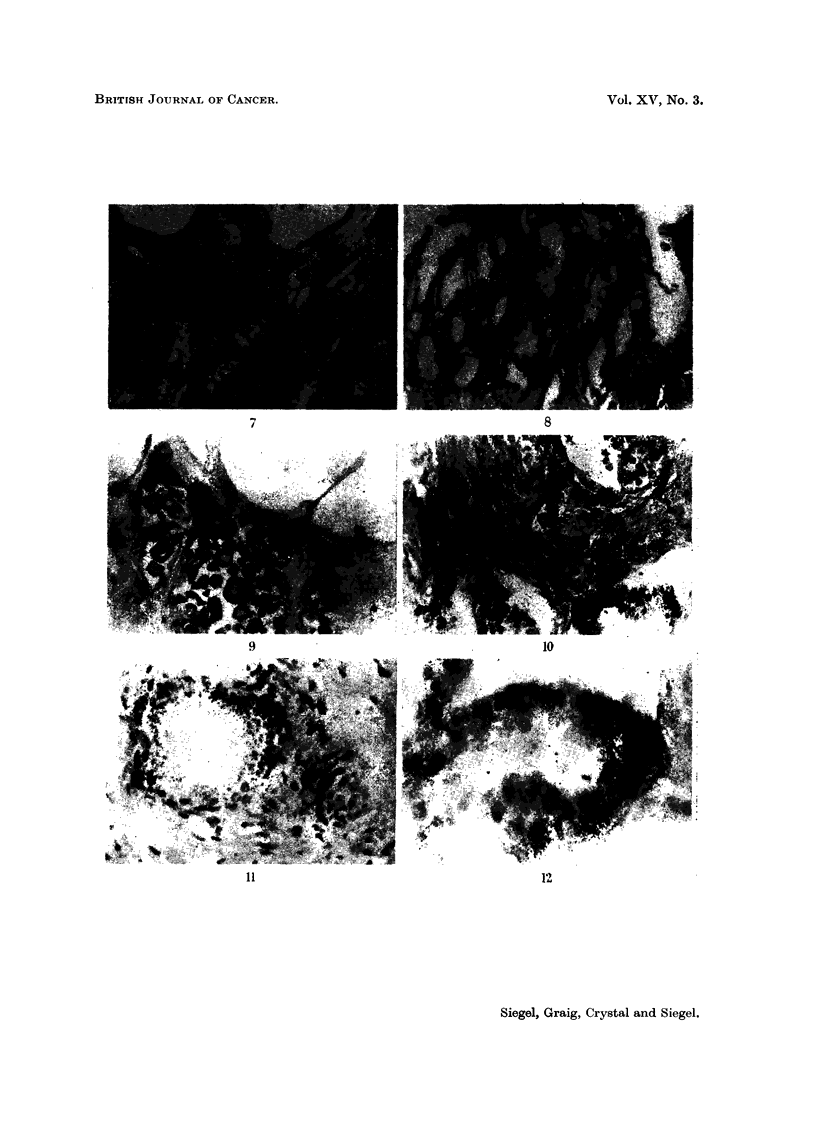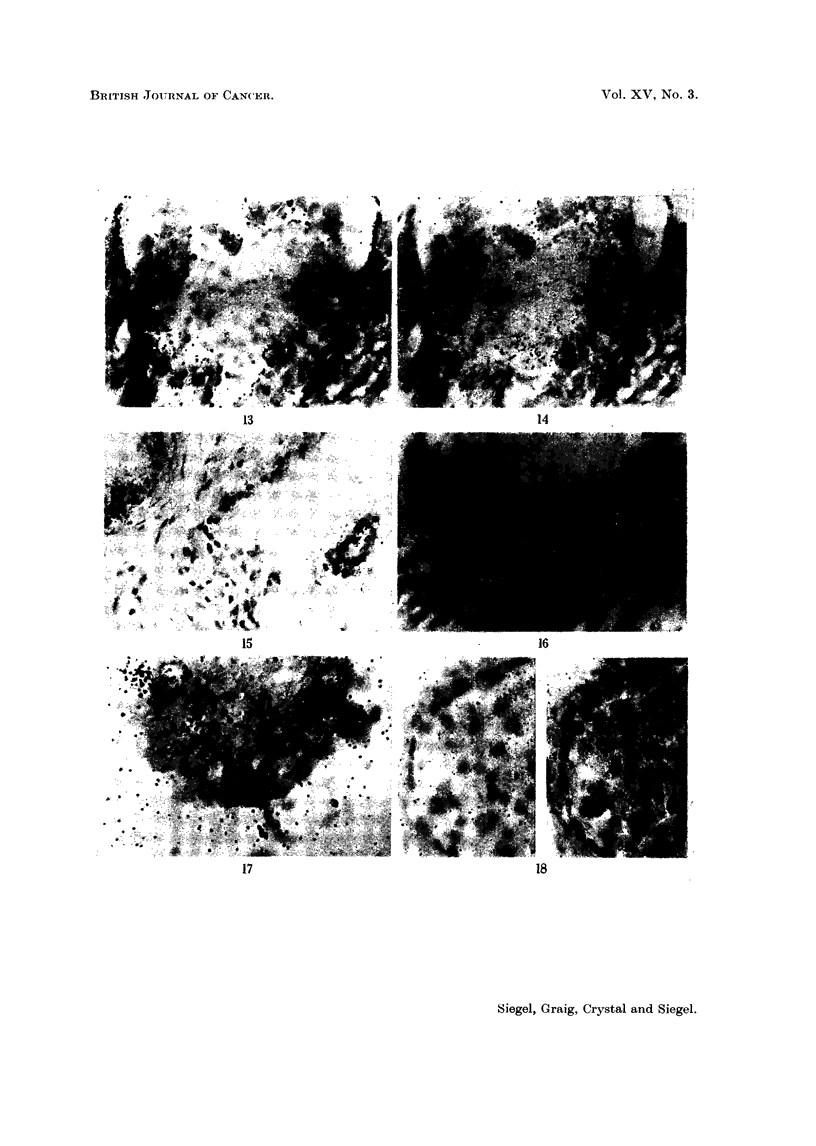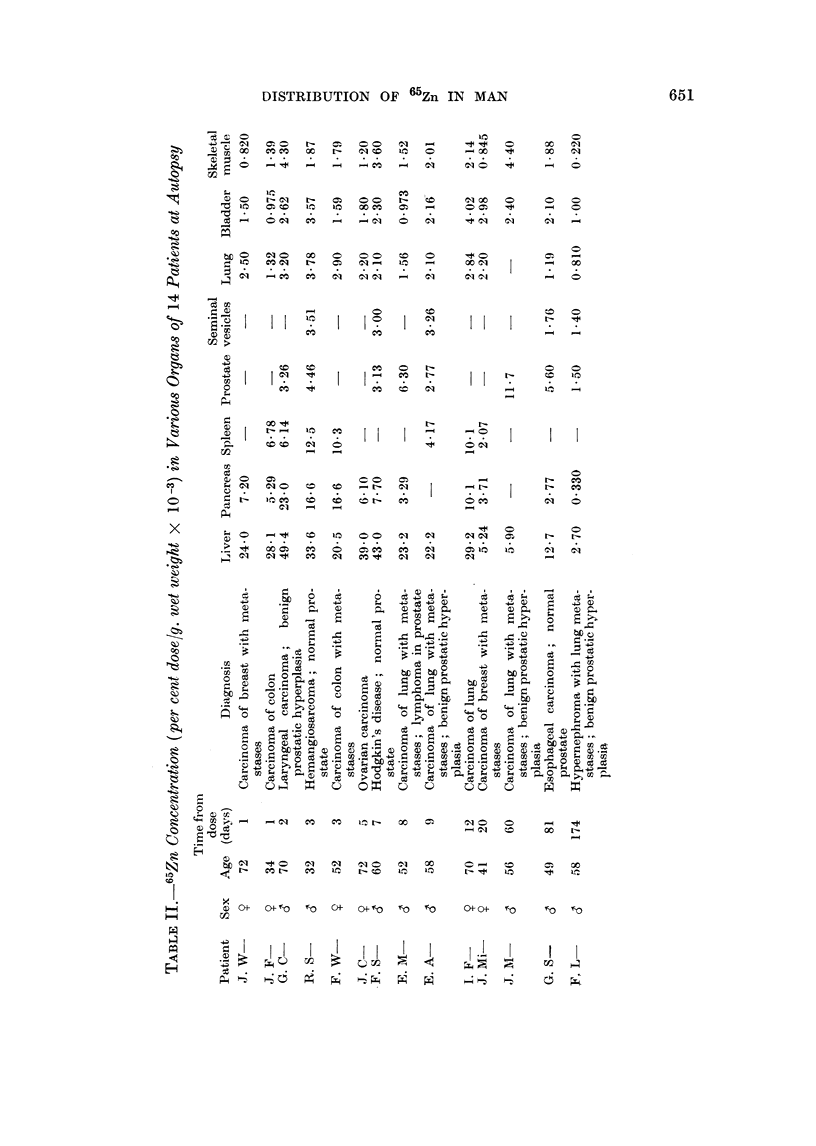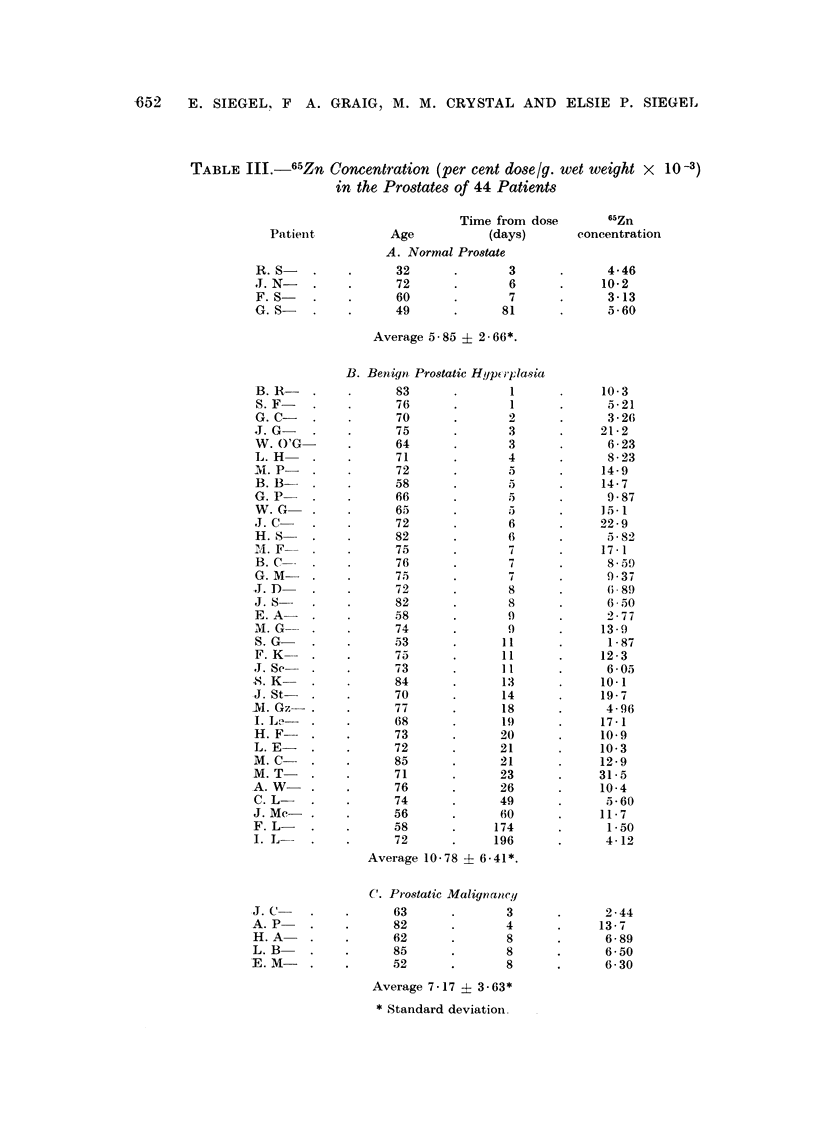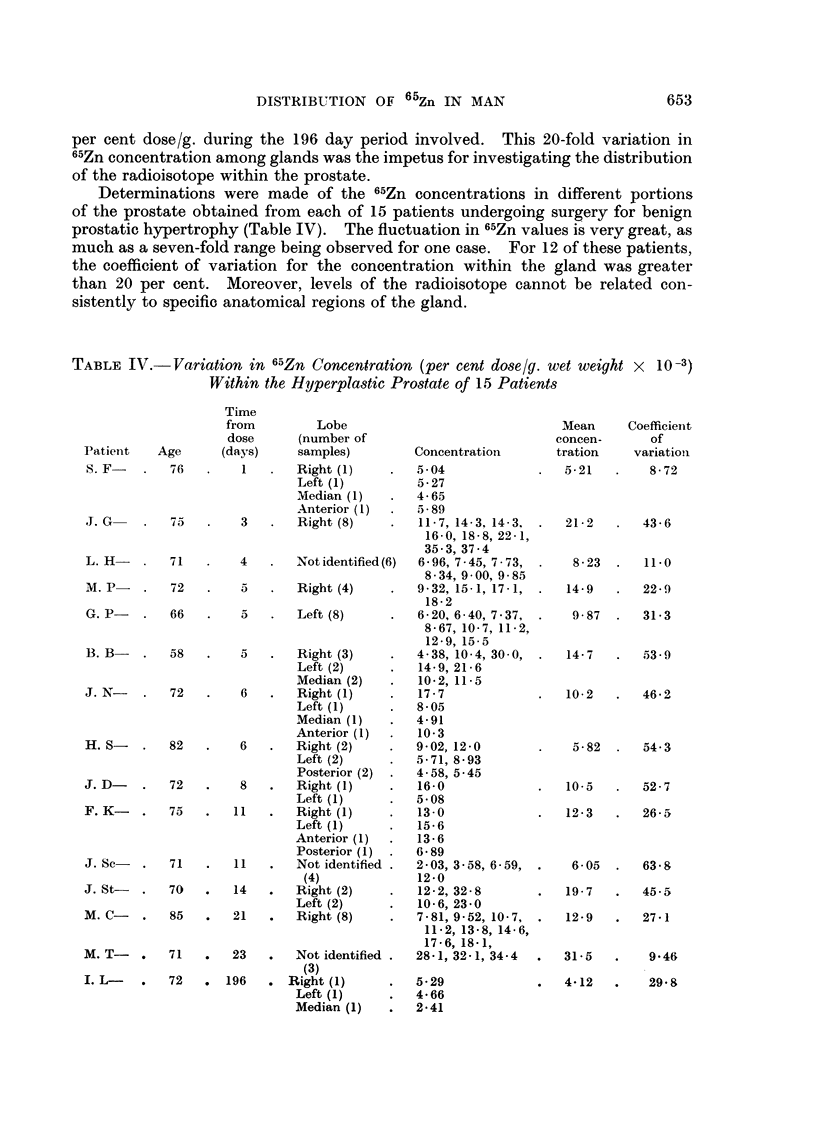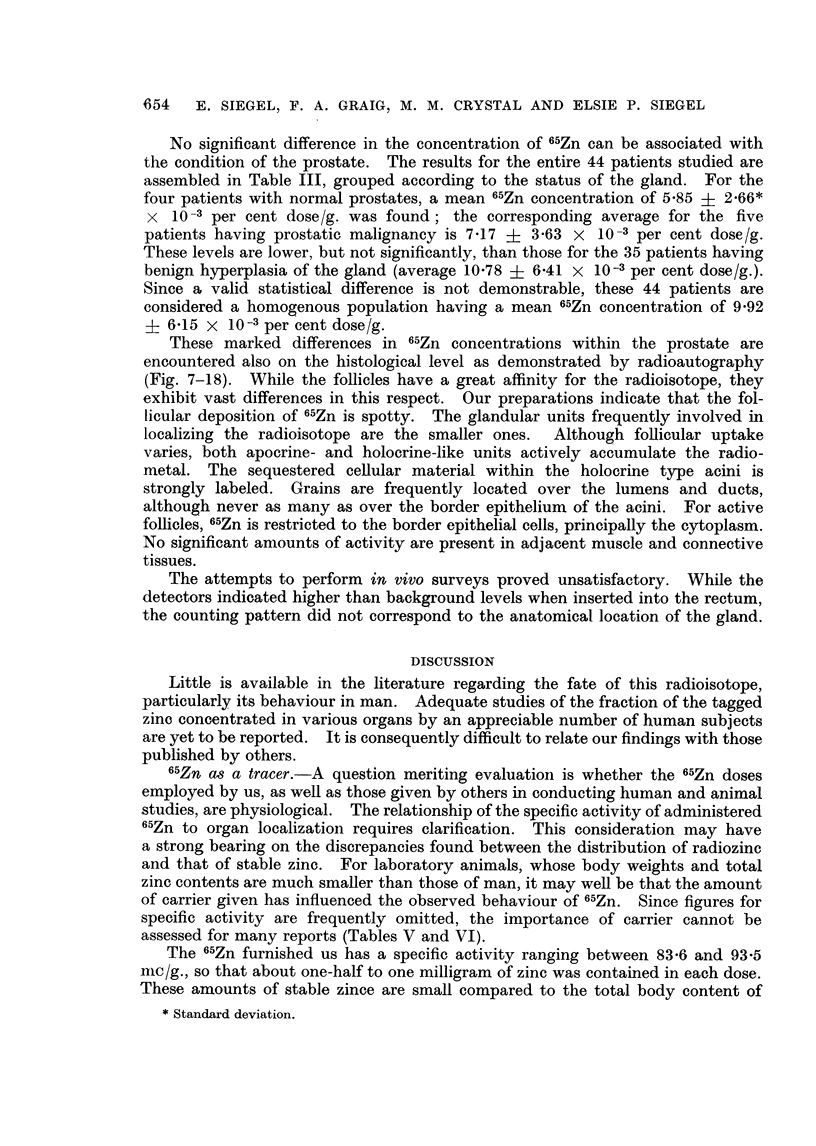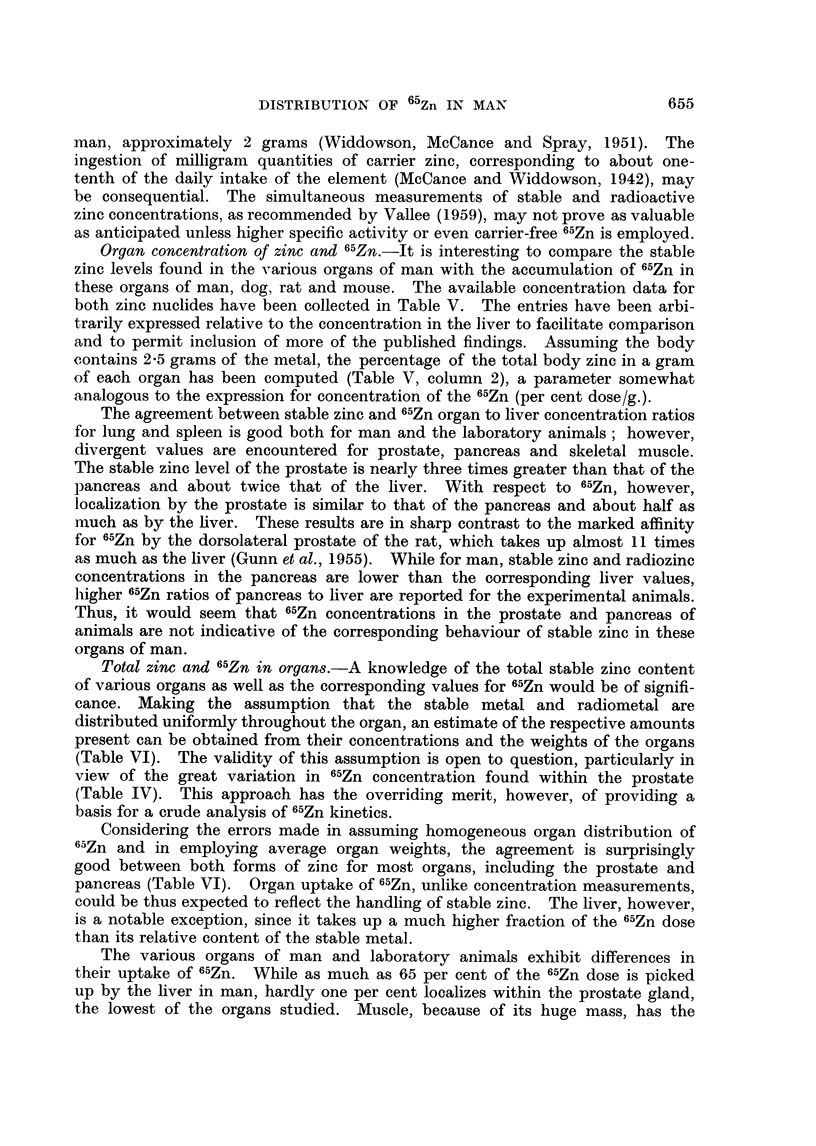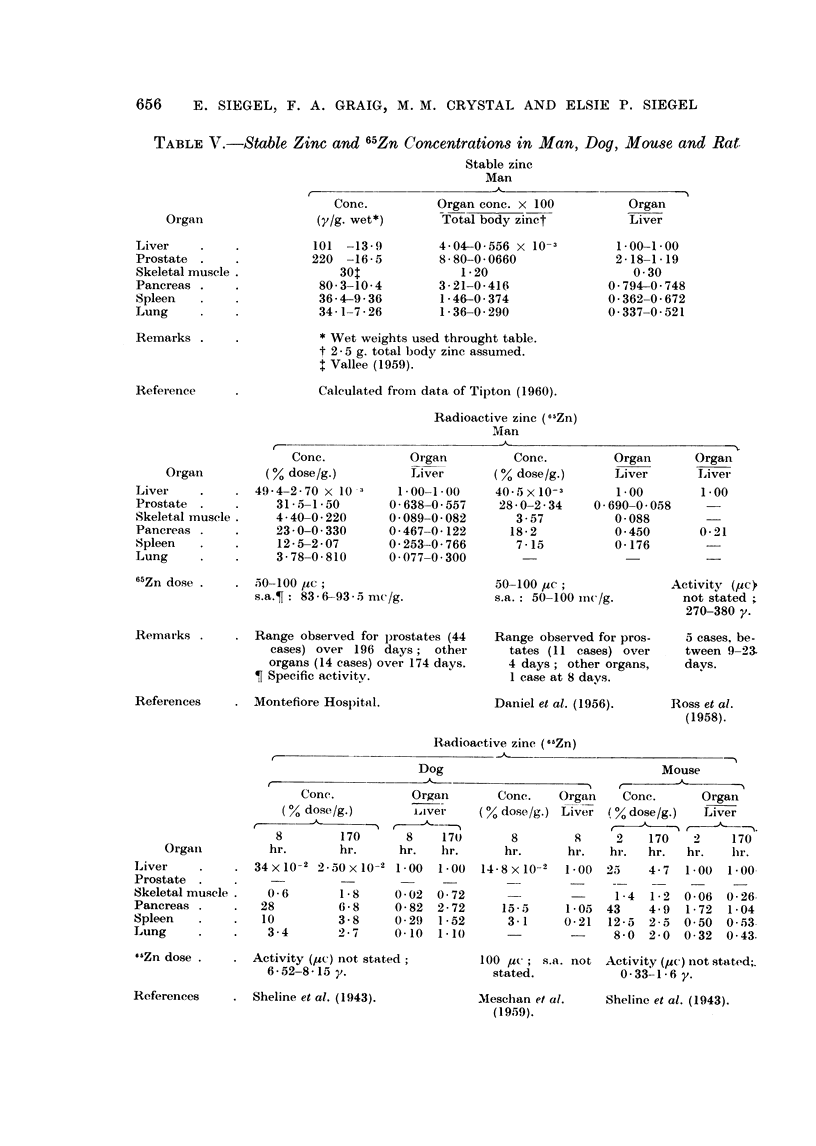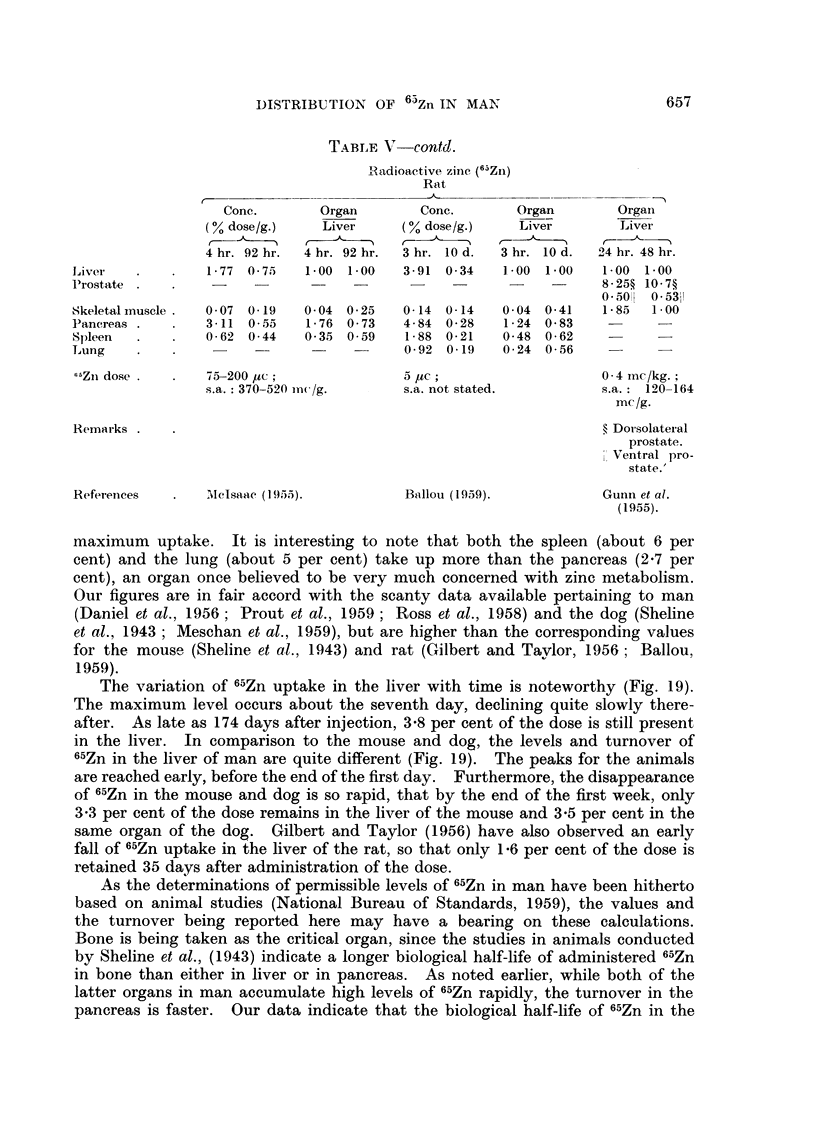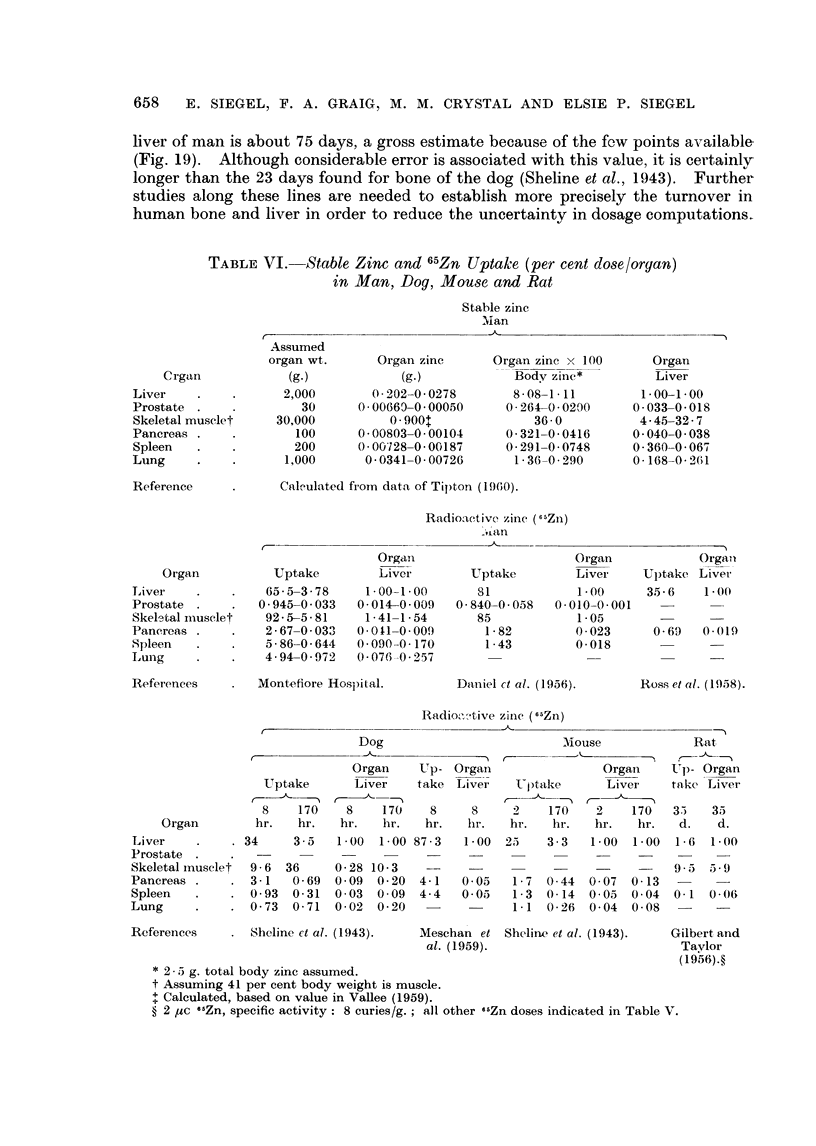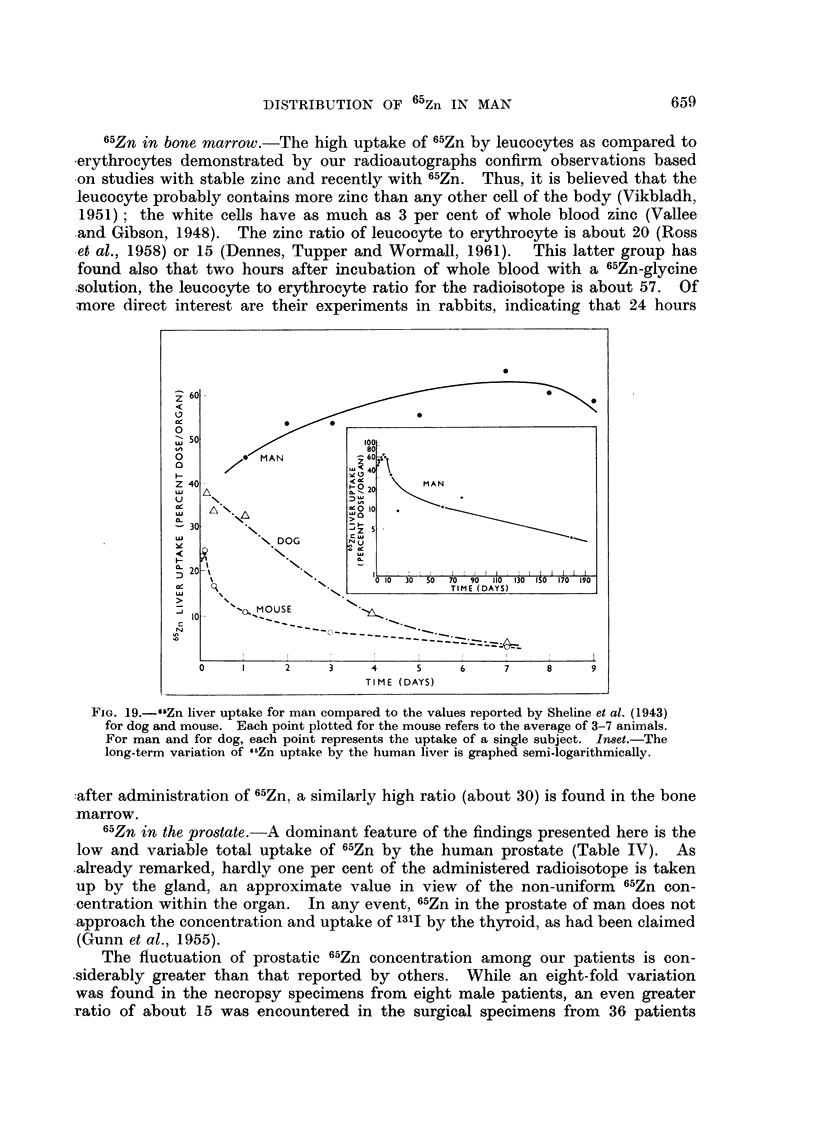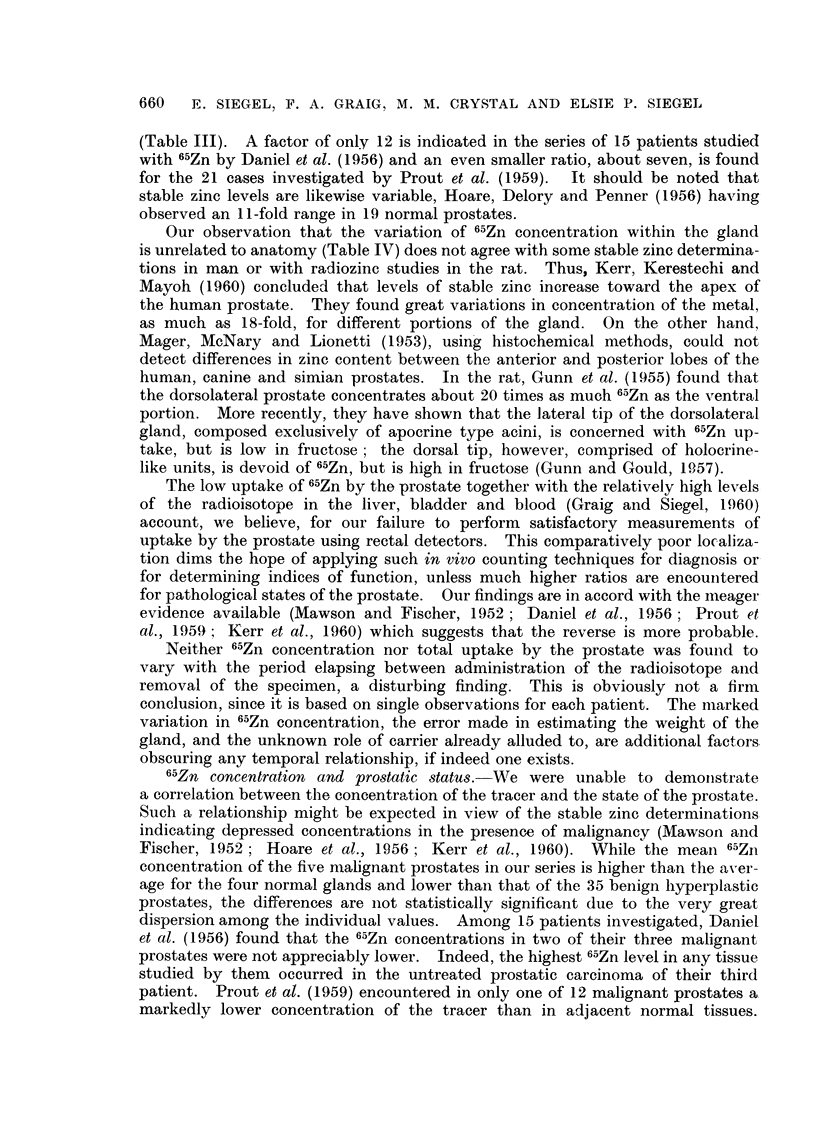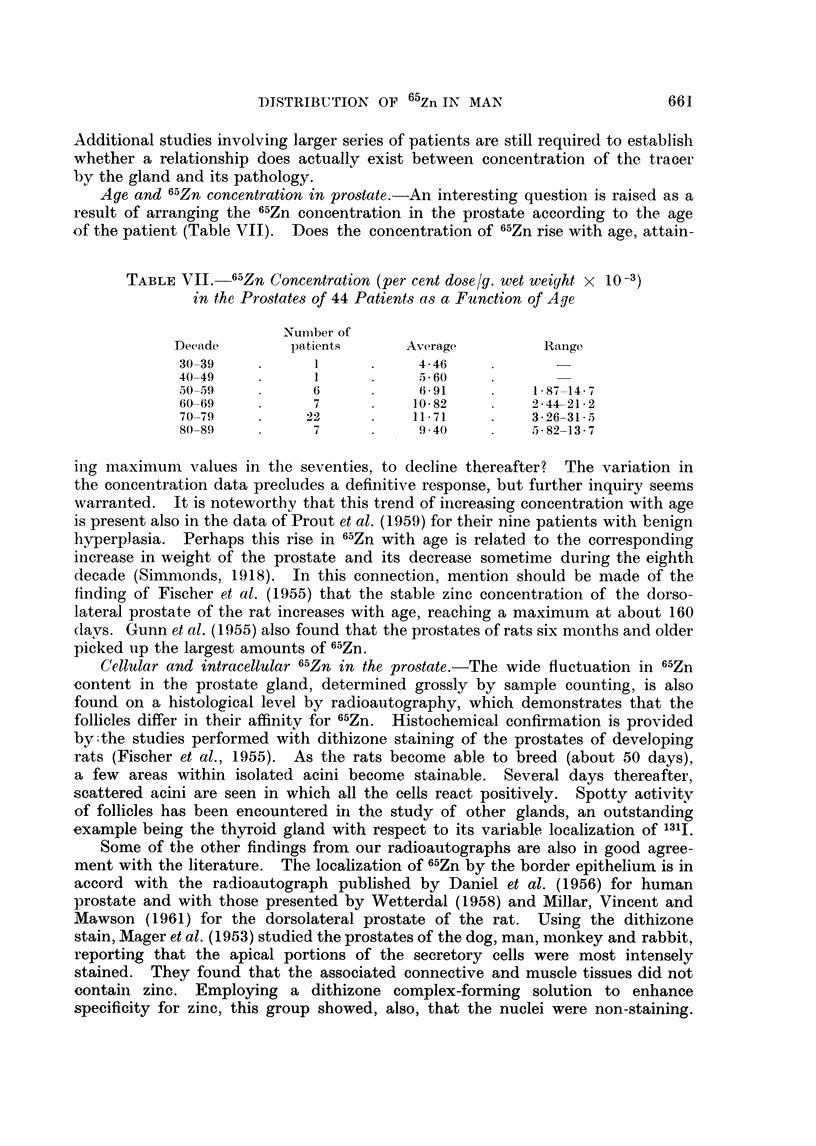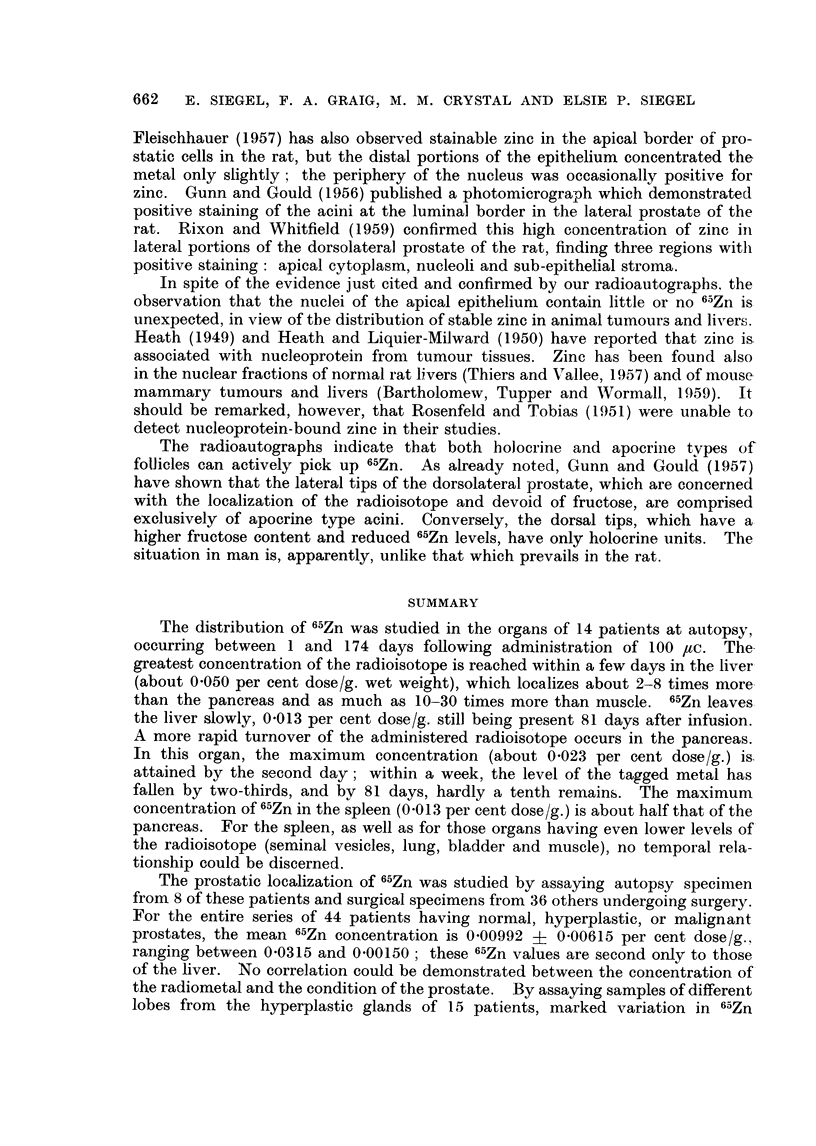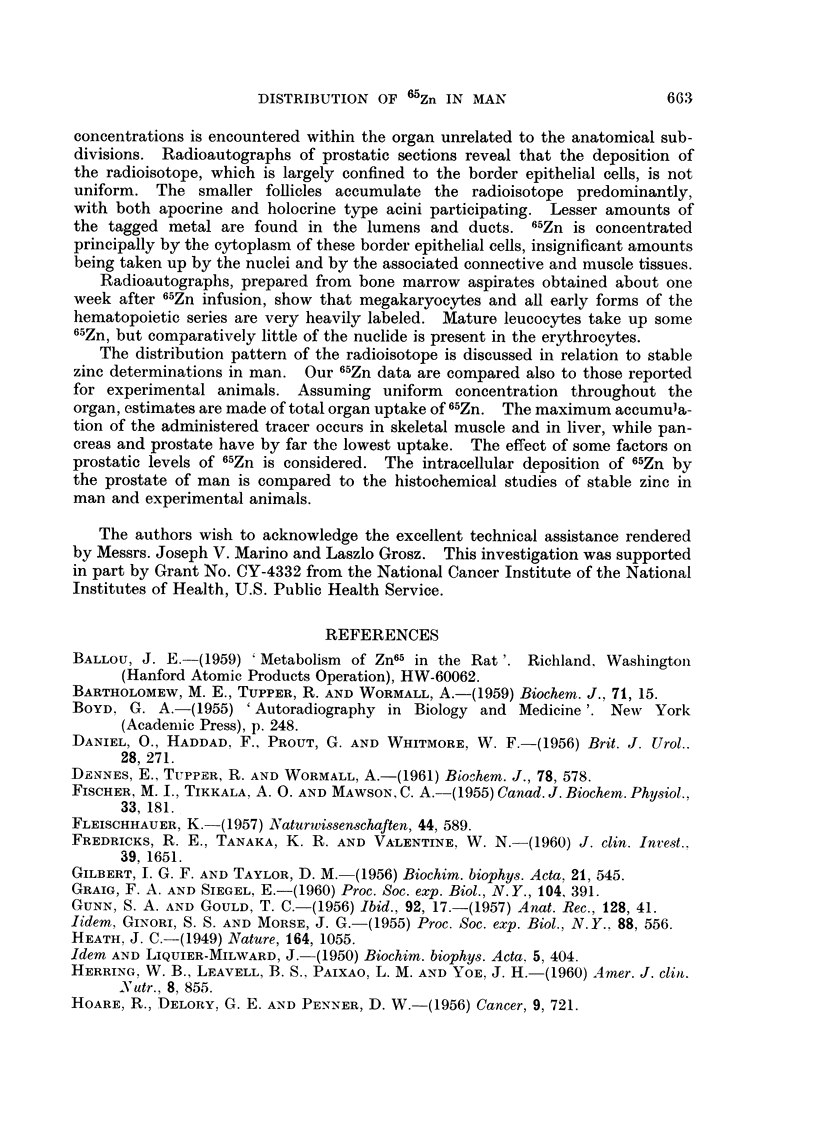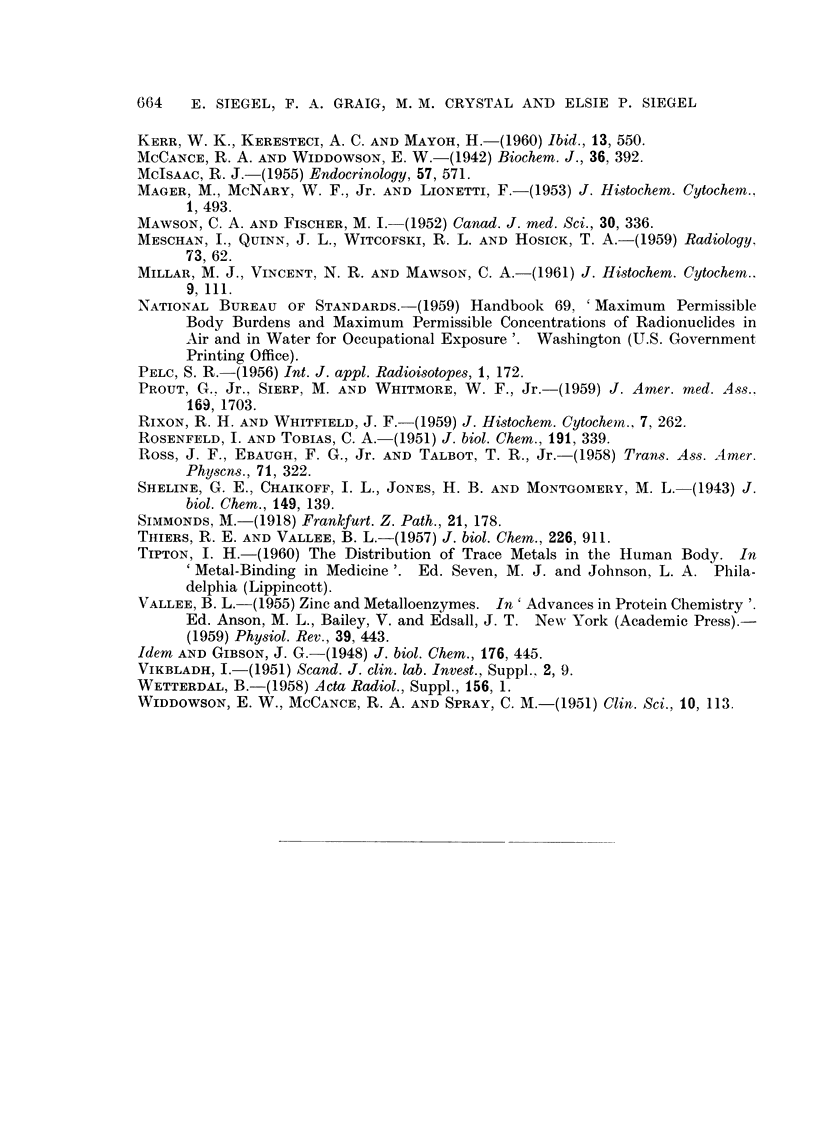# Distribution of ^65^Zn in the Prostate and other Organs of Man

**DOI:** 10.1038/bjc.1961.74

**Published:** 1961-09

**Authors:** E. Siegel, F. A. Graig, M. M. Crystal, Elsie P. Siegel

## Abstract

**Images:**


### DISTRIBUTION OF $^{65}Zn$ IN THE PROSTATE AND OTHER ORGANS OF MAN

E. SIEGEL, F. A. GRAIG, M. M. CRYSTAL AND ELSIE P. SIEGEL

*From the Medical Physics Laboratory, Medical Division, Montefiore Hospital, New York* 67, *N.Y., U.S.A.*

Received for publication July 1, 1961

ALTHOUGH there are many biological studies dealing with zinc, little quantiative data have appeared regarding the concentration and turnover of this metal, especially for man. Interest in zinc metabolism has been stimulated by the accumulating evidence which indicates that this metal is a constituent of several enzymes (Fischer, Tikkala and Mawson, 1955 ; Vallee, 1955) and is associated, also, with some pathological states (Daniel *et al.*, 1956 ; Prout, Sierp and Whitmore, 1959 ; Herring *et al.*, 1961 ; Fredricks, Tanaka and Valentine, 1960). Our concern with the element stems from reports of the high concentration of zinc found in the prostate gland both in animals (Gunn *et al.*, 1955) and man (Mawson and Fischer, 1952).

While its biological significance is established, the physiological role of zinc remains to be elucidated. Important contributions to our understanding of the part played by the element should result from investigations which employ $^{65}Zn$. Precise data are not yet available as to the fraction of the administered radioisotope which is taken up by the various organs. Quantitative concentration data are unavailable for the prostate ; moreover, the particular sites and structures of the gland associated with $^{65}Zn$ uptake have not been identified.

Following our earlier study of the distribution of $^{65}Zn$ in blood and its excretion in man (Graig and Siegel, 1960), the present investigation was undertaken to learn more about the concentration of the radioisotope by several organs with special attention to the prostate. For the latter, it was also sought to ascertain which glandular components are implicated in the accumulation of $^{65}Zn$.

### MATERIALS AND METHODS

*Autopsy studies.*—Fourteen pre-terminal patients having various malignancies were given approximately 100 $\mu c$ $^{65}Zn$ (specific activity greater than 75 mc/g.) as the chloride. The radioisotope was administered as an intravenous infusion in a 250 ml. volume of isotonic solution. At autopsy, which occurred between 1 and 174 days after the infusion, portions of the following organs were obtained, whenever possible, for digestion and the assay of $^{65}Zn$ : liver, pancreas, spleen, prostate, seminal vesicles, lung, bladder and skeletal muscle.

*Studies on prostatic surgical specimens.*—Tracer doses of $^{65}Zn$ (approximately 50 $\mu c$) were administered in the manner described above to 36 patients presumed to have diseased prostates and awaiting surgery. A sample of each surgical specimen was assayed subsequently for $^{65}Zn$ concentration, using the technique outlined below. The variaton of radiozinc localization within the prostate itself was investigated in 15 of these patients having benign hypertrophy. Multiple samples were taken from the different lobes (left, right, medial, anterior and posterior) of the gland for determination of $^{65}Zn$.

*Digestion of organs.*—Duplicate weighed samples of each specimen were covered and digested with 20 per cent NaOH in a chemical hood employing moderate heat for several hours. Throughout this period, the level of liquid was maintained by the addition of distilled water. To determine $^{65}Zn$ concentration (per cent dose/g.), aliquots were taken from the resulting suspension for assay.

Recovery experiments were performed on freshly killed mouse organs to check the reliability of this digestion technique. Known activities of $^{65}Zn$ (6·3 $\mu$c contained in 1 ml.) were added to duplicate samples of each of the following animal organs: brain, lung, spleen, heart, liver, pancreas, kidney, adrenal, muscle and skin. As indicated in Table I, the results justified employing this method for the processing of the various human samples.

TABLE I.—*Results of $^{65}Zn$ Recovery Experiments, Following NaOH Digestion of Various Mouse Organs with Added $^{65}Zn$*

| Organ | Per cent $^{65}Zn$ recovered |
|---|---|
| Adrenal | 99·5 |
| Brain | 104·3 |
| Heart | 100·5 |
| Kidney | 101·5 |
| Liver | 99·5 |
| Lung | 100·0 |
| Muscle | 100·0 |
| Pancreas | 99·5 |
| Spleen | 100·5 |
| Skin | 99·5 |
| | 100·5 ± 1·6* |

* Standard deviation.

*Assay of $^{65}Zn$.*—Counting of organ suspensions was performed with a well-type scintillation counter, having a 1½ inch diameter NaI-thallium activated crystal. This detector has a sensitivity of $2{\cdot}92 \times 10^5$ cpm/$\mu$c for $^{65}Zn$, established by calibration against National Bureau of Standards reference solutions. Corrections for physical decay of $^{65}Zn$ (half-life 244·4 days) were made either from long term decay curves or from concurrent measurements of aliquots of dose solutions. Adequate counts were collected to assure an accuracy of better than 5 per cent.

IN VIVO *counting of prostate.*—The localization of $^{65}Zn$ by the intact prostate was attempted by using shielded rectal detectors possessing high gamma sensitivity. An Antol type 302 Geiger counter was inserted into a 4 mm. thick lead housing, whose external diameter was 17·5 mm. With a 1 cm. port at the distal end, this counting arrangement had a sensitivity of 100 cpm/$\mu$c of $^{65}Zn$ in contact with a source whose volume was 1 ml. More recently, a Nuclear-Chicago DS-8 scintillation probe was used similarly; its sensitivity with a 6 mm. diameter needle probe was 6700 cpm/$\mu$c in contact.

*Radioautography*

(a) *Prostatic sections.*—To study the localization of the administered radioisotope, radioautographs were prepared from 5–10 micron sections of prostate specimens obtained either at surgery or at autopsy. The organ samples were fixed overnight in absolute methyl alcohol to minimize loss of $^{65}$Zn and then cleared in xylene. Unstained sections were coated with Kodak Ltd. AR-10 stripping film (Pelc, 1956), exposed for periods ranging between 14 and 250 days at 4°C. in the presence of a desiccant, after which they were developed for 8 minutes in D-19 developer at 18°C. Following fixing and washing, the radioautographs were stained either with hematoxylin and eosin or with methyl green-pyronin. To check for artifacts, control radioautographs were prepared from non-radioactive prostatic sections.

(b) *Bone marrow smears.*—About a week after the infusion of $^{65}$Zn tracer doses, bone marrow aspirates from ten patients having no known hematological disorders were smeared in the darkroom on Kodak NTB plates and fixed with absolute methyl alcohol (Boyd, 1955). Following exposure for over 200 days and photographic processing, the plates were stained with Giemsa.

### RESULTS

*Distribution of $^{65}$Zn in various organs and tissues*

Widely different concentrations of radiozinc were found in the organs obtained at autopsy (Table II). The highest levels are those of the liver, reaching about 0·050 per cent dose/g. (wet weight) within a few days following administration of the tagged metal. These values for the concentration in the liver are about 2–8 times greater than those in the pancreas, the next most active organ, and as much as 10–30 times the levels in muscle, the tissue with the lowest concentration in our study. Turnover of $^{65}$Zn in the liver is apparently slow ; as late as 81 days following infusion of the radioisotope, the concentration is still about 0·013 per cent dose/g. The radioisotope is handled more rapidly by the pancreas, so that the maximum concentration, about 0·023 per cent dose/g., is attained by the second day. Within a week, the $^{65}$Zn level of the pancreas is reduced by two-thirds, and by 81 days, hardly a tenth remains. The maximum $^{65}$Zn concentration in the spleen, about half that of the pancreas, occurred on the third day. The small amounts of radiozinc in the seminal vesicles, bladder and muscle do not appear to change significantly with time.

The radioautographs prepared from the bone marrow smears (Fig. 1–6) indicate that the greatest amounts of $^{65}$Zn are taken up by all early forms of the hematopoietic series. Megakaryocytes are also very heavily labeled. Mature leucocytes take up lesser amounts of the radioisotope. Since virtually no grains appear over the erythrocytes, they must contain comparatively little $^{65}$Zn. No difference between the accumulation of radiozinc in nucleus and cytoplasm of the leucocyte can be discerned.

*$^{65}$Zn concentration by the prostate*

Among the organs assayed at autopsy, the prostate holds intermediate rank with respect to its ability to concentrate $^{65}$Zn (Table II). For the eight males so studied, the mean $^{65}$Zn concentration is 0·00495 per cent dose/g. (wet weight)

with values ranging between 0·00150 and 0·0117. Compared to the other organs, the ability of the gland to take up administered $^{65}Zn$ is very variable ; it is also not related to time.

This finding of widely scattered radiozinc values independent of the interval following the infusion of the tracer is reinforced by the measurements made on the surgical specimens of the prostate obtained from 36 patients (Table III). For all 44 male patients, the concentrations ranged between 0·00150 and 0·0315

### EXPLANATION OF PLATES

FIG. 1–5.—Patient M. K—. Radioautograph of bone marrow smear, prepared 8 days following administration of 100 $\mu c$ $^{65}Zn$, 215-day exposure. Giemsa.

FIG. 1.—Note large number of grains over early forms, fewer over leucocytes and virtual absence of grains over erythrocytes. × 180.

FIG. 2.—Megakaryocyte demonstrating marked $^{65}Zn$ localization. × 405.

FIG. 3.—Normoblast showing very high concentration of $^{65}Zn$. × 405.

FIG. 4.—Myelocyte indicating considerable uptake of radiozinc. × 405.

FIG. 5.—Note moderate $^{65}Zn$ uptake in the 4 neutrophils. × 405.

FIG. 6.—Patient F. S—. Radioautograph of bone marrow smear prepared 6 days following administration of 50 $\mu c$ $^{65}Zn$. The metamyelocyte is heavily labeled with $^{65}Zn$. 205-day exposure. Giemsa. × 405.

FIG. 7.—Patient M. F— (benign prostatic hyperplasia). Radioautograph of prostatic section from surgical specimen obtained 7 days after administration of 50 $\mu c$ $^{65}Zn$. Distribution of the radioisotope is spotty and largely confined to the border epithelial cells of the smaller acini. 61-day exposure. Methyl green-pyronin. × 42.

FIG. 8.—Patient L. E— (benign prostatic hyperplasia). Radioautograph of prostatic section from surgical specimen obtained 21 days after administration of 50 $\mu c$ $^{65}Zn$. Parts of several small acini are labeled with radiozinc. 119-day exposure. Methyl green-pyronin. × 42.

FIG. 9.—M. F— (same as Fig. 7—different section). Maximum $^{65}Zn$ localization is along the luminal border. The prostatic concretions are not as heavily labeled. 136-day exposure. Methyl green-pyronin. × 42.

FIG. 10.—Patient L. E— (same as Fig. 8). One of three adjacent follicles is very actively concentrating the radioisotope. The surrounding stroma does not pick up $^{65}Zn$ appreciably. Photographic grains appear principally over the border epithelium. × 180.

FIG. 11.—Patient C. L— (benign prostatic hyperplasia). Radioautograph of prostatic section from surgical specimen obtained 49 days after administration of 50 $\mu c$ $^{65}Zn$. Uptake by the follicle is not uniform. 65-day exposure. H. and E. × 180.

FIG. 12.—Patient L. E— (same as Fig. 8—different section). Variable distribution of $^{65}Zn$ in border epithelium of acinus. Methyl green-pyronin. × 405.

FIG. 13.—Patient L. E— (same as Fig. 8). Microscope focused on photographic grains over luminal border. × 405.

FIG. 14.—Patient L. E—. Same field and magnification as Fig. 13, focused on photographic grains over lumen.

FIG. 15.—Patient C. L— (same as Fig. 11). Sequestered cellular material in the holocrine type acinus is labeled as well as the border epithelial cells of this and the neighboring follicle. × 180.

FIG. 16.—Patient M. F— (same as Fig. 7). $^{65}Zn$ is taken up predominantly by the cytoplasm of the border epithelial cells. Relatively few grains appear over the adjacent stroma. × 405.

FIG. 17.—Patient M. F— (same as Fig. 7). Another field demonstrating the localization of $^{65}Zn$ in the cytoplasm of the acinar epithelium. Lesser amounts of tagged material are found in the lumen. × 405.

FIG. 18.—Patient E. Mc— (lymphoma in prostate). Radioautograph of prostatic section from necropsy specimen obtained 9 days after administration of 50 $\mu c$ $^{65}Zn$. Left : focus is on grains ; right: focus is on the plane of the cells. Note that the radioisotope is generally outside the nucleus. 47-day exposure. Methyl green-pyronin. × 405.

1 2 3 4 5 6

Siegel, Graig, Crystal and Siegel.

7

8

9

10

11

12

13

14

15

16

17

18

Siegel, Graig, Crystal and Siegel.

TABLE II.—$^{65}$*Zn Concentration* (*per cent dose/g. wet weight* × $10^{-3}$) *in Various Organs of* 14 *Patients at Autopsy*

| Patient | Sex | Age | Time from dose (days) | Diagnosis | Liver | Pancreas | Spleen | Prostate | Seminal vesicles | Lung | Bladder | Skeletal muscle |
|---|---|---|---|---|---|---|---|---|---|---|---|---|
| J. W— | ♀ | 72 | 1 | Carcinoma of breast with metastases | 24·0 | 7·20 | — | — | — | 2·50 | 1·50 | 0·820 |
| J. F— | ♀ | 34 | 1 | Carcinoma of colon | 28·1 | 5·29 | 6·78 | — | — | 1·32 | 0·975 | 1·39 |
| G. C— | ♂ | 70 | 2 | Laryngeal carcinoma; benign prostatic hyperplasia | 49·4 | 23·0 | 6·14 | 3·26 | — | 3·20 | 2·62 | 4·30 |
| R. S— | ♂ | 32 | 3 | Hemangiosarcoma; normal prostate | 33·6 | 16·6 | 12·5 | 4·46 | 3·51 | 3·78 | 3·57 | 1·87 |
| F. W— | ♀ | 52 | 3 | Carcinoma of colon with metastases | 20·5 | 16·6 | 10·3 | — | — | 2·90 | 1·59 | 1·79 |
| J. C— | ♀ | 72 | 5 | Ovarian carcinoma | 39·0 | 6·10 | — | — | — | 2·20 | 1·80 | 1·20 |
| F. S— | ♂ | 60 | 7 | Hodgkin's disease; normal prostate | 43·0 | 7·70 | — | 3·13 | 3·00 | 2·10 | 2·30 | 3·60 |
| E. M— | ♂ | 52 | 8 | Carcinoma of lung with metastases; lymphoma in prostate | 23·2 | 3·29 | — | 6·30 | — | 1·56 | 0·973 | 1·52 |
| E. A— | ♂ | 58 | 9 | Carcinoma of lung with metastases; benign prostatic hyperplasia | 22·2 | — | 4·17 | 2·77 | 3·26 | 2·10 | 2·16 | 2·01 |
| I. F— | ♀ | 70 | 12 | Carcinoma of lung | 29·2 | 10·1 | 10·1 | — | — | 2·84 | 4·02 | 2·14 |
| J. Mi— | ♀ | 41 | 20 | Carcinoma of breast with metastases | 5·24 | 3·71 | 2·07 | — | — | 2·20 | 2·98 | 0·845 |
| J. M— | ♂ | 56 | 60 | Carcinoma of lung with metastases; benign prostatic hyperplasia | 5·90 | — | — | 11·7 | — | — | 2·40 | 4·40 |
| G. S— | ♂ | 49 | 81 | Esophageal carcinoma; normal prostate | 12·7 | 2·77 | — | 5·60 | 1·76 | 1·19 | 2·10 | 1·88 |
| F. L— | ♂ | 58 | 174 | Hypernephroma with lung metastases; benign prostatic hyperplasia | 2·70 | 0·330 | — | 1·50 | 1·40 | 0·810 | 1·00 | 0·220 |

TABLE III.—$^{65}$*Zn Concentration* (*per cent dose/g. wet weight* $\times 10^{-3}$) *in the Prostates of* 44 *Patients*

| Patient | Age | Time from dose (days) | $^{65}$Zn concentration |
|---|---|---|---|
| | *A. Normal Prostate* | | |
| R. S— | 32 | 3 | 4·46 |
| J. N— | 72 | 6 | 10·2 |
| F. S— | 60 | 7 | 3·13 |
| G. S— | 49 | 81 | 5·60 |
| | Average 5·85 ± 2·66*. | | |
| | *B. Benign Prostatic Hyperplasia* | | |
| B. R— | 83 | 1 | 10·3 |
| S. F— | 76 | 1 | 5·21 |
| G. C— | 70 | 2 | 3·26 |
| J. G— | 75 | 3 | 21·2 |
| W. O'G— | 64 | 3 | 6·23 |
| L. H— | 71 | 4 | 8·23 |
| M. P— | 72 | 5 | 14·9 |
| B. B— | 58 | 5 | 14·7 |
| G. P— | 66 | 5 | 9·87 |
| W. G— | 65 | 5 | 15·1 |
| J. C— | 72 | 6 | 22·9 |
| H. S— | 82 | 6 | 5·82 |
| M. F— | 75 | 7 | 17·1 |
| B. C— | 76 | 7 | 8·59 |
| G. M— | 75 | 7 | 9·37 |
| J. D— | 72 | 8 | 6·89 |
| J. S— | 82 | 8 | 6·50 |
| E. A— | 58 | 9 | 2·77 |
| M. G— | 74 | 9 | 13·9 |
| S. G— | 53 | 11 | 1·87 |
| F. K— | 75 | 11 | 12·3 |
| J. Sc— | 73 | 11 | 6·05 |
| S. K— | 84 | 13 | 10·1 |
| J. St— | 70 | 14 | 19·7 |
| M. Gz— | 77 | 18 | 4·96 |
| I. Le— | 68 | 19 | 17·1 |
| H. F— | 73 | 20 | 10·9 |
| L. E— | 72 | 21 | 10·3 |
| M. C— | 85 | 21 | 12·9 |
| M. T— | 71 | 23 | 31·5 |
| A. W— | 76 | 26 | 10·4 |
| C. L— | 74 | 49 | 5·60 |
| J. Mc— | 56 | 60 | 11·7 |
| F. L— | 58 | 174 | 1·50 |
| I. L— | 72 | 196 | 4·12 |
| | Average 10·78 ± 6·41*. | | |
| | *C. Prostatic Malignancy* | | |
| J. C— | 63 | 3 | 2·44 |
| A. P— | 82 | 4 | 13·7 |
| H. A— | 62 | 8 | 6·89 |
| L. B— | 85 | 8 | 6·50 |
| E. M— | 52 | 8 | 6·30 |
| | Average 7·17 ± 3·63* | | |

* Standard deviation.

per cent dose/g. during the 196 day period involved. This 20-fold variation in $^{65}Zn$ concentration among glands was the impetus for investigating the distribution of the radioisotope within the prostate.

Determinations were made of the $^{65}Zn$ concentrations in different portions of the prostate obtained from each of 15 patients undergoing surgery for benign prostatic hypertrophy (Table IV). The fluctuation in $^{65}Zn$ values is very great, as much as a seven-fold range being observed for one case. For 12 of these patients, the coefficient of variation for the concentration within the gland was greater than 20 per cent. Moreover, levels of the radioisotope cannot be related consistently to specific anatomical regions of the gland.

TABLE IV.—*Variation in $^{65}Zn$ Concentration (per cent dose/g. wet weight $\times$ $10^{-3}$) Within the Hyperplastic Prostate of* 15 *Patients*

| Patient | Age | Time from dose (days) | Lobe (number of samples) | Concentration | Mean concentration | Coefficient of variation |
|---|---|---|---|---|---|---|
| S. F— | 76 | 1 | Right (1) | 5·04 | 5·21 | 8·72 |
| | | | Left (1) | 5·27 | | |
| | | | Median (1) | 4·65 | | |
| | | | Anterior (1) | 5·89 | | |
| J. G— | 75 | 3 | Right (8) | 11·7, 14·3, 14·3, 16·0, 18·8, 22·1, 35·3, 37·4 | 21·2 | 43·6 |
| L. H— | 71 | 4 | Not identified (6) | 6·96, 7·45, 7·73, 8·34, 9·00, 9·85 | 8·23 | 11·0 |
| M. P— | 72 | 5 | Right (4) | 9·32, 15·1, 17·1, 18·2 | 14·9 | 22·9 |
| G. P— | 66 | 5 | Left (8) | 6·20, 6·40, 7·37, 8·67, 10·7, 11·2, 12·9, 15·5 | 9·87 | 31·3 |
| B. B— | 58 | 5 | Right (3) | 4·38, 10·4, 30·0, | 14·7 | 53·9 |
| | | | Left (2) | 14·9, 21·6 | | |
| | | | Median (2) | 10·2, 11·5 | | |
| J. N— | 72 | 6 | Right (1) | 17·7 | 10·2 | 46·2 |
| | | | Left (1) | 8·05 | | |
| | | | Median (1) | 4·91 | | |
| | | | Anterior (1) | 10·3 | | |
| H. S— | 82 | 6 | Right (2) | 9·02, 12·0 | 5·82 | 54·3 |
| | | | Left (2) | 5·71, 8·93 | | |
| | | | Posterior (2) | 4·58, 5·45 | | |
| J. D— | 72 | 8 | Right (1) | 16·0 | 10·5 | 52·7 |
| | | | Left (1) | 5·08 | | |
| F. K— | 75 | 11 | Right (1) | 13·0 | 12·3 | 26·5 |
| | | | Left (1) | 15·6 | | |
| | | | Anterior (1) | 13·6 | | |
| | | | Posterior (1) | 6·89 | | |
| J. Sc— | 71 | 11 | Not identified (4) | 2·03, 3·58, 6·59, 12·0 | 6·05 | 63·8 |
| J. St— | 70 | 14 | Right (2) | 12·2, 32·8 | 19·7 | 45·5 |
| | | | Left (2) | 10·6, 23·0 | | |
| M. C— | 85 | 21 | Right (8) | 7·81, 9·52, 10·7, 11·2, 13·8, 14·6, 17·6, 18·1, | 12·9 | 27·1 |
| M. T— | 71 | 23 | Not identified (3) | 28·1, 32·1, 34·4 | 31·5 | 9·46 |
| I. L— | 72 | 196 | Right (1) | 5·29 | 4·12 | 29·8 |
| | | | Left (1) | 4·66 | | |
| | | | Median (1) | 2·41 | | |

No significant difference in the concentration of $^{65}Zn$ can be associated with the condition of the prostate. The results for the entire 44 patients studied are assembled in Table III, grouped according to the status of the gland. For the four patients with normal prostates, a mean $^{65}Zn$ concentration of $5 \cdot 85 \pm 2 \cdot 66$* $\times 10^{-3}$ per cent dose/g. was found; the corresponding average for the five patients having prostatic malignancy is $7 \cdot 17 \pm 3 \cdot 63 \times 10^{-3}$ per cent dose/g. These levels are lower, but not significantly, than those for the 35 patients having benign hyperplasia of the gland (average $10 \cdot 78 \pm 6 \cdot 41 \times 10^{-3}$ per cent dose/g.). Since a valid statistical difference is not demonstrable, these 44 patients are considered a homogenous population having a mean $^{65}Zn$ concentration of $9 \cdot 92 \pm 6 \cdot 15 \times 10^{-3}$ per cent dose/g.

These marked differences in $^{65}Zn$ concentrations within the prostate are encountered also on the histological level as demonstrated by radioautography (Fig. 7–18). While the follicles have a great affinity for the radioisotope, they exhibit vast differences in this respect. Our preparations indicate that the follicular deposition of $^{65}Zn$ is spotty. The glandular units frequently involved in localizing the radioisotope are the smaller ones. Although follicular uptake varies, both apocrine- and holocrine-like units actively accumulate the radiometal. The sequestered cellular material within the holocrine type acini is strongly labeled. Grains are frequently located over the lumens and ducts, although never as many as over the border epithelium of the acini. For active follicles, $^{65}Zn$ is restricted to the border epithelial cells, principally the cytoplasm. No significant amounts of activity are present in adjacent muscle and connective tissues.

The attempts to perform *in vivo* surveys proved unsatisfactory. While the detectors indicated higher than background levels when inserted into the rectum, the counting pattern did not correspond to the anatomical location of the gland.

### DISCUSSION

Little is available in the literature regarding the fate of this radioisotope, particularly its behaviour in man. Adequate studies of the fraction of the tagged zinc concentrated in various organs by an appreciable number of human subjects are yet to be reported. It is consequently difficult to relate our findings with those published by others.

*$^{65}Zn$ as a tracer.*—A question meriting evaluation is whether the $^{65}Zn$ doses employed by us, as well as those given by others in conducting human and animal studies, are physiological. The relationship of the specific activity of administered $^{65}Zn$ to organ localization requires clarification. This consideration may have a strong bearing on the discrepancies found between the distribution of radiozinc and that of stable zinc. For laboratory animals, whose body weights and total zinc contents are much smaller than those of man, it may well be that the amount of carrier given has influenced the observed behaviour of $^{65}Zn$. Since figures for specific activity are frequently omitted, the importance of carrier cannot be assessed for many reports (Tables V and VI).

The $^{65}Zn$ furnished us has a specific activity ranging between 83·6 and 93·5 mc/g., so that about one-half to one milligram of zinc was contained in each dose. These amounts of stable zince are small compared to the total body content of

* Standard deviation.

man, approximately 2 grams (Widdowson, McCance and Spray, 1951). The ingestion of milligram quantities of carrier zinc, corresponding to about one-tenth of the daily intake of the element (McCance and Widdowson, 1942), may be consequential. The simultaneous measurements of stable and radioactive zinc concentrations, as recommended by Vallee (1959), may not prove as valuable as anticipated unless higher specific activity or even carrier-free $^{65}$Zn is employed.

*Organ concentration of zinc and $^{65}$Zn.*—It is interesting to compare the stable zinc levels found in the various organs of man with the accumulation of $^{65}$Zn in these organs of man, dog, rat and mouse. The available concentration data for both zinc nuclides have been collected in Table V. The entries have been arbitrarily expressed relative to the concentration in the liver to facilitate comparison and to permit inclusion of more of the published findings. Assuming the body contains 2·5 grams of the metal, the percentage of the total body zinc in a gram of each organ has been computed (Table V, column 2), a parameter somewhat analogous to the expression for concentration of the $^{65}$Zn (per cent dose/g.).

The agreement between stable zinc and $^{65}$Zn organ to liver concentration ratios for lung and spleen is good both for man and the laboratory animals ; however, divergent values are encountered for prostate, pancreas and skeletal muscle. The stable zinc level of the prostate is nearly three times greater than that of the pancreas and about twice that of the liver. With respect to $^{65}$Zn, however, localization by the prostate is similar to that of the pancreas and about half as much as by the liver. These results are in sharp contrast to the marked affinity for $^{65}$Zn by the dorsolateral prostate of the rat, which takes up almost 11 times as much as the liver (Gunn *et al.*, 1955). While for man, stable zinc and radiozinc concentrations in the pancreas are lower than the corresponding liver values, higher $^{65}$Zn ratios of pancreas to liver are reported for the experimental animals. Thus, it would seem that $^{65}$Zn concentrations in the prostate and pancreas of animals are not indicative of the corresponding behaviour of stable zinc in these organs of man.

*Total zinc and $^{65}$Zn in organs.*—A knowledge of the total stable zinc content of various organs as well as the corresponding values for $^{65}$Zn would be of significance. Making the assumption that the stable metal and radiometal are distributed uniformly throughout the organ, an estimate of the respective amounts present can be obtained from their concentrations and the weights of the organs (Table VI). The validity of this assumption is open to question, particularly in view of the great variation in $^{65}$Zn concentration found within the prostate (Table IV). This approach has the overriding merit, however, of providing a basis for a crude analysis of $^{65}$Zn kinetics.

Considering the errors made in assuming homogeneous organ distribution of $^{65}$Zn and in employing average organ weights, the agreement is surprisingly good between both forms of zinc for most organs, including the prostate and pancreas (Table VI). Organ uptake of $^{65}$Zn, unlike concentration measurements, could be thus expected to reflect the handling of stable zinc. The liver, however, is a notable exception, since it takes up a much higher fraction of the $^{65}$Zn dose than its relative content of the stable metal.

The various organs of man and laboratory animals exhibit differences in their uptake of $^{65}$Zn. While as much as 65 per cent of the $^{65}$Zn dose is picked up by the liver in man, hardly one per cent localizes within the prostate gland, the lowest of the organs studied. Muscle, because of its huge mass, has the

TABLE V.—*Stable Zinc and* $^{65}$*Zn Concentrations in Man, Dog, Mouse and Rat*

Stable zinc — Man

| Organ | Conc. (γ/g. wet*) | Organ conc. × 100 / Total body zinc† | Organ / Liver |
|---|---|---|---|
| Liver | 101 –13·9 | $4·04–0·556 \times 10^{-3}$ | 1·00–1·00 |
| Prostate | 220 –16·5 | 8·80–0·0660 | 2·18–1·19 |
| Skeletal muscle | 30‡ | 1·20 | 0·30 |
| Pancreas | 80·3–10·4 | 3·21–0·416 | 0·794–0·748 |
| Spleen | 36·4–9·36 | 1·46–0·374 | 0·362–0·672 |
| Lung | 34·1–7·26 | 1·36–0·290 | 0·337–0·521 |
| Remarks | * Wet weights used throught table.<br>† 2·5 g. total body zinc assumed.<br>‡ Vallee (1959). | | |
| Reference | Calculated from data of Tipton (1960). | | |

Radioactive zinc ($^{65}$Zn) — Man

| Organ | Conc. (% dose/g.) | Organ / Liver | Conc. (% dose/g.) | Organ / Liver | Organ / Liver |
|---|---|---|---|---|---|
| Liver | $49·4–2·70 \times 10^{-3}$ | 1·00–1·00 | $40·5 \times 10^{-3}$ | 1·00 | 1·00 |
| Prostate | 31·5–1·50 | 0·638–0·557 | 28·0–2·34 | 0·690–0·058 | — |
| Skeletal muscle | 4·40–0·220 | 0·089–0·082 | 3·57 | 0·088 | — |
| Pancreas | 23·0–0·330 | 0·467–0·122 | 18·2 | 0·450 | 0·21 |
| Spleen | 12·5–2·07 | 0·253–0·766 | 7·15 | 0·176 | — |
| Lung | 3·78–0·810 | 0·077–0·300 | — | — | — |
| $^{65}$Zn dose | 50–100 μc ; s.a.¶ : 83·6–93·5 mc/g. | | 50–100 μc ; s.a. : 50–100 mc/g. | | Activity (μc) not stated ; 270–380 γ. |
| Remarks | Range observed for prostates (44 cases) over 196 days ; other organs (14 cases) over 174 days. ¶ Specific activity. | | Range observed for prostates (11 cases) over 4 days ; other organs, 1 case at 8 days. | | 5 cases, between 9–23 days. |
| References | Montefiore Hospital. | | Daniel *et al.* (1956). | | Ross *et al.* (1958). |

Radioactive zinc ($^{65}$Zn)

| Organ | Dog: Conc. (% dose/g.) 8 hr. | Dog: Conc. (% dose/g.) 170 hr. | Dog: Organ/Liver 8 hr. | Dog: Organ/Liver 170 hr. | Dog: Conc. (% dose/g.) 8 hr. | Dog: Organ/Liver 8 hr. | Mouse: Conc. (% dose/g.) 2 hr. | Mouse: Conc. (% dose/g.) 170 hr. | Mouse: Organ/Liver 2 hr. | Mouse: Organ/Liver 170 hr. |
|---|---|---|---|---|---|---|---|---|---|---|
| Liver | $34 \times 10^{-2}$ | $2·50 \times 10^{-2}$ | 1·00 | 1·00 | $14·8 \times 10^{-2}$ | 1·00 | 25 | 4·7 | 1·00 | 1·00 |
| Prostate | — | — | — | — | — | — | — | — | — | — |
| Skeletal muscle | 0·6 | 1·8 | 0·02 | 0·72 | — | — | 1·4 | 1·2 | 0·06 | 0·26 |
| Pancreas | 28 | 6·8 | 0·82 | 2·72 | 15·5 | 1·05 | 43 | 4·9 | 1·72 | 1·04 |
| Spleen | 10 | 3·8 | 0·29 | 1·52 | 3·1 | 0·21 | 12·5 | 2·5 | 0·50 | 0·53 |
| Lung | 3·4 | 2·7 | 0·10 | 1·10 | — | — | 8·0 | 2·0 | 0·32 | 0·43 |
| $^{65}$Zn dose | Activity (μc) not stated ; 6·52–8·15 γ. | | | | 100 μc ; s.a. not stated. | | Activity (μc) not stated; 0·33–1·6 γ. | | | |
| References | Sheline *et al.* (1943). | | | | Meschan *et al.* (1959). | | Sheline *et al.* (1943). | | | |

TABLE V—*contd.*

| | Radioactive zinc ($^{65}$Zn) Rat | | | | | | | | | |
|---|---|---|---|---|---|---|---|---|---|---|
| | Conc. (% dose/g.) | | Organ/Liver | | Conc. (% dose/g.) | | Organ/Liver | | Organ/Liver | |
| | 4 hr. | 92 hr. | 4 hr. | 92 hr. | 3 hr. | 10 d. | 3 hr. | 10 d. | 24 hr. | 48 hr. |
| Liver | 1·77 | 0·75 | 1·00 | 1·00 | 3·91 | 0·34 | 1·00 | 1·00 | 1·00 | 1·00 |
| Prostate | — | — | — | — | — | — | — | — | 8·25§<br>0·50‖ | 10·7§<br>0·53‖ |
| Skeletal muscle | 0·07 | 0·19 | 0·04 | 0·25 | 0·14 | 0·14 | 0·04 | 0·41 | 1·85 | 1·00 |
| Pancreas | 3·11 | 0·55 | 1·76 | 0·73 | 4·84 | 0·28 | 1·24 | 0·83 | — | — |
| Spleen | 0·62 | 0·44 | 0·35 | 0·59 | 1·88 | 0·21 | 0·48 | 0·62 | — | — |
| Lung | — | — | — | — | 0·92 | 0·19 | 0·24 | 0·56 | — | — |
| $^{65}$Zn dose | 75–200 μc ; s.a. : 370–520 mc/g. | | | | 5 μc ; s.a. not stated. | | | | 0·4 mc/kg. ; s.a. : 120–164 mc/g. | |
| Remarks | | | | | | | | | § Dorsolateral prostate. ‖ Ventral prostate.' | |
| References | McIsaac (1955). | | | | Ballou (1959). | | | | Gunn *et al.* (1955). | |

maximum uptake. It is interesting to note that both the spleen (about 6 per cent) and the lung (about 5 per cent) take up more than the pancreas (2·7 per cent), an organ once believed to be very much concerned with zinc metabolism. Our figures are in fair accord with the scanty data available pertaining to man (Daniel *et al.*, 1956 ; Prout *et al.*, 1959 ; Ross *et al.*, 1958) and the dog (Sheline *et al.*, 1943 ; Meschan *et al.*, 1959), but are higher than the corresponding values for the mouse (Sheline *et al.*, 1943) and rat (Gilbert and Taylor, 1956 ; Ballou, 1959).

The variation of $^{65}$Zn uptake in the liver with time is noteworthy (Fig. 19). The maximum level occurs about the seventh day, declining quite slowly thereafter. As late as 174 days after injection, 3·8 per cent of the dose is still present in the liver. In comparison to the mouse and dog, the levels and turnover of $^{65}$Zn in the liver of man are quite different (Fig. 19). The peaks for the animals are reached early, before the end of the first day. Furthermore, the disappearance of $^{65}$Zn in the mouse and dog is so rapid, that by the end of the first week, only 3·3 per cent of the dose remains in the liver of the mouse and 3·5 per cent in the same organ of the dog. Gilbert and Taylor (1956) have also observed an early fall of $^{65}$Zn uptake in the liver of the rat, so that only 1·6 per cent of the dose is retained 35 days after administration of the dose.

As the determinations of permissible levels of $^{65}$Zn in man have been hitherto based on animal studies (National Bureau of Standards, 1959), the values and the turnover being reported here may have a bearing on these calculations. Bone is being taken as the critical organ, since the studies in animals conducted by Sheline *et al.*, (1943) indicate a longer biological half-life of administered $^{65}$Zn in bone than either in liver or in pancreas. As noted earlier, while both of the latter organs in man accumulate high levels of $^{65}$Zn rapidly, the turnover in the pancreas is faster. Our data indicate that the biological half-life of $^{65}$Zn in the liver of man is about 75 days, a gross estimate because of the few points available (Fig. 19). Although considerable error is associated with this value, it is certainly longer than the 23 days found for bone of the dog (Sheline *et al.*, 1943). Further studies along these lines are needed to establish more precisely the turnover in human bone and liver in order to reduce the uncertainty in dosage computations.

TABLE VI.—*Stable Zinc and $^{65}$Zn Uptake (per cent dose/organ) in Man, Dog, Mouse and Rat*

| Organ | Stable zinc, Man: Assumed organ wt. (g.) | Organ zinc (g.) | Organ zinc × 100 / Body zinc* | Organ / Liver |
|---|---|---|---|---|
| Liver | 2,000 | 0·202–0·0278 | 8·08–1·11 | 1·00–1·00 |
| Prostate | 30 | 0·00660–0·00050 | 0·264–0·0200 | 0·033–0·018 |
| Skeletal muscle† | 30,000 | 0·900‡ | 36·0 | 4·45–32·7 |
| Pancreas | 100 | 0·00803–0·00104 | 0·321–0·0416 | 0·040–0·038 |
| Spleen | 200 | 0·00728–0·00187 | 0·291–0·0748 | 0·360–0·067 |
| Lung | 1,000 | 0·0341–0·00726 | 1·36–0·290 | 0·168–0·261 |
| Reference | Calculated from data of Tipton (1960). | | | |

| Organ | Radioactive zinc ($^{65}$Zn), Man: Uptake | Organ / Liver | Uptake | Organ / Liver | Uptake | Organ / Liver |
|---|---|---|---|---|---|---|
| Liver | 65·5–3·78 | 1·00–1·00 | 81 | 1·00 | 35·6 | 1·00 |
| Prostate | 0·945–0·033 | 0·014–0·009 | 0·840–0·058 | 0·010–0·001 | — | — |
| Skeletal muscle† | 92·5–5·81 | 1·41–1·54 | 85 | 1·05 | — | — |
| Pancreas | 2·67–0·033 | 0·041–0·009 | 1·82 | 0·023 | 0·69 | 0·019 |
| Spleen | 5·86–0·644 | 0·090–0·170 | 1·43 | 0·018 | — | — |
| Lung | 4·94–0·972 | 0·076–0·257 | — | — | — | — |
| References | Montefiore Hospital. | | Daniel *et al.* (1956). | | Ross *et al.* (1958). | |

| | Radioactive zinc ($^{65}$Zn) | | | | | | | | | | | | |
|---|---|---|---|---|---|---|---|---|---|---|---|---|---|
| | Dog | | | | | | Mouse | | | | Rat | |
| | Uptake | | Organ / Liver | | Up-take | Organ / Liver | Uptake | | Organ / Liver | | Up-take | Organ / Liver |
| Organ | 8 hr. | 170 hr. | 8 hr. | 170 hr. | 8 hr. | 8 hr. | 2 hr. | 170 hr. | 2 hr. | 170 hr. | 35 d. | 35 d. |
| Liver | 34 | 3·5 | 1·00 | 1·00 | 87·3 | 1·00 | 25 | 3·3 | 1·00 | 1·00 | 1·6 | 1·00 |
| Prostate | — | — | — | — | — | — | — | — | — | — | — | — |
| Skeletal muscle† | 9·6 | 36 | 0·28 | 10·3 | — | — | — | — | — | — | 9·5 | 5·9 |
| Pancreas | 3·1 | 0·69 | 0·09 | 0·20 | 4·1 | 0·05 | 1·7 | 0·44 | 0·07 | 0·13 | — | — |
| Spleen | 0·93 | 0·31 | 0·03 | 0·09 | 4·4 | 0·05 | 1·3 | 0·14 | 0·05 | 0·04 | 0·1 | 0·06 |
| Lung | 0·73 | 0·71 | 0·02 | 0·20 | — | — | 1·1 | 0·26 | 0·04 | 0·08 | — | — |
| References | Sheline *et al.* (1943). | | | | Meschan *et al.* (1959). | | Sheline *et al.* (1943). | | | | Gilbert and Taylor (1956).§ | |

* 2·5 g. total body zinc assumed.
† Assuming 41 per cent body weight is muscle.
‡ Calculated, based on value in Vallee (1959).
§ 2 μc $^{65}$Zn, specific activity : 8 curies/g. ; all other $^{65}$Zn doses indicated in Table V.

$^{65}$*Zn in bone marrow.*—The high uptake of $^{65}$Zn by leucocytes as compared to erythrocytes demonstrated by our radioautographs confirm observations based on studies with stable zinc and recently with $^{65}$Zn. Thus, it is believed that the leucocyte probably contains more zinc than any other cell of the body (Vikbladh, 1951); the white cells have as much as 3 per cent of whole blood zinc (Vallee and Gibson, 1948). The zinc ratio of leucocyte to erythrocyte is about 20 (Ross *et al.*, 1958) or 15 (Dennes, Tupper and Wormall, 1961). This latter group has found also that two hours after incubation of whole blood with a $^{65}$Zn-glycine solution, the leucocyte to erythrocyte ratio for the radioisotope is about 57. Of more direct interest are their experiments in rabbits, indicating that 24 hours

FIG. 19.—$^{65}$Zn liver uptake for man compared to the values reported by Sheline *et al.* (1943) for dog and mouse. Each point plotted for the mouse refers to the average of 3–7 animals. For man and for dog, each point represents the uptake of a single subject. *Inset.*—The long-term variation of $^{65}$Zn uptake by the human liver is graphed semi-logarithmically.

after administration of $^{65}$Zn, a similarly high ratio (about 30) is found in the bone marrow.

$^{65}$*Zn in the prostate.*—A dominant feature of the findings presented here is the low and variable total uptake of $^{65}$Zn by the human prostate (Table IV). As already remarked, hardly one per cent of the administered radioisotope is taken up by the gland, an approximate value in view of the non-uniform $^{65}$Zn concentration within the organ. In any event, $^{65}$Zn in the prostate of man does not approach the concentration and uptake of $^{131}$I by the thyroid, as had been claimed (Gunn *et al.*, 1955).

The fluctuation of prostatic $^{65}$Zn concentration among our patients is considerably greater than that reported by others. While an eight-fold variation was found in the necropsy specimens from eight male patients, an even greater ratio of about 15 was encountered in the surgical specimens from 36 patients

(Table III). A factor of only 12 is indicated in the series of 15 patients studied with $^{65}$Zn by Daniel *et al.* (1956) and an even smaller ratio, about seven, is found for the 21 cases investigated by Prout *et al.* (1959). It should be noted that stable zinc levels are likewise variable, Hoare, Delory and Penner (1956) having observed an 11-fold range in 19 normal prostates.

Our observation that the variation of $^{65}$Zn concentration within the gland is unrelated to anatomy (Table IV) does not agree with some stable zinc determinations in man or with radiozinc studies in the rat. Thus, Kerr, Kerestechi and Mayoh (1960) concluded that levels of stable zinc increase toward the apex of the human prostate. They found great variations in concentration of the metal, as much as 18-fold, for different portions of the gland. On the other hand, Mager, McNary and Lionetti (1953), using histochemical methods, could not detect differences in zinc content between the anterior and posterior lobes of the human, canine and simian prostates. In the rat, Gunn *et al.* (1955) found that the dorsolateral prostate concentrates about 20 times as much $^{65}$Zn as the ventral portion. More recently, they have shown that the lateral tip of the dorsolateral gland, composed exclusively of apocrine type acini, is concerned with $^{65}$Zn uptake, but is low in fructose ; the dorsal tip, however, comprised of holocrine-like units, is devoid of $^{65}$Zn, but is high in fructose (Gunn and Gould, 1957).

The low uptake of $^{65}$Zn by the prostate together with the relatively high levels of the radioisotope in the liver, bladder and blood (Graig and Siegel, 1960) account, we believe, for our failure to perform satisfactory measurements of uptake by the prostate using rectal detectors. This comparatively poor localization dims the hope of applying such *in vivo* counting techniques for diagnosis or for determining indices of function, unless much higher ratios are encountered for pathological states of the prostate. Our findings are in accord with the meager evidence available (Mawson and Fischer, 1952 ; Daniel *et al.*, 1956 ; Prout *et al.*, 1959 ; Kerr *et al.*, 1960) which suggests that the reverse is more probable.

Neither $^{65}$Zn concentration nor total uptake by the prostate was found to vary with the period elapsing between administration of the radioisotope and removal of the specimen, a disturbing finding. This is obviously not a firm conclusion, since it is based on single observations for each patient. The marked variation in $^{65}$Zn concentration, the error made in estimating the weight of the gland, and the unknown role of carrier already alluded to, are additional factors obscuring any temporal relationship, if indeed one exists.

*$^{65}$Zn concentration and prostatic status.*—We were unable to demonstrate a correlation between the concentration of the tracer and the state of the prostate. Such a relationship might be expected in view of the stable zinc determinations indicating depressed concentrations in the presence of malignancy (Mawson and Fischer, 1952 ; Hoare *et al.*, 1956 ; Kerr *et al.*, 1960). While the mean $^{65}$Zn concentration of the five malignant prostates in our series is higher than the average for the four normal glands and lower than that of the 35 benign hyperplastic prostates, the differences are not statistically significant due to the very great dispersion among the individual values. Among 15 patients investigated, Daniel *et al.* (1956) found that the $^{65}$Zn concentrations in two of their three malignant prostates were not appreciably lower. Indeed, the highest $^{65}$Zn level in any tissue studied by them occurred in the untreated prostatic carcinoma of their third patient. Prout *et al.* (1959) encountered in only one of 12 malignant prostates a markedly lower concentration of the tracer than in adjacent normal tissues.

Additional studies involving larger series of patients are still required to establish whether a relationship does actually exist between concentration of the tracer by the gland and its pathology.

*Age and $^{65}$Zn concentration in prostate.*—An interesting question is raised as a result of arranging the $^{65}$Zn concentration in the prostate according to the age of the patient (Table VII). Does the concentration of $^{65}$Zn rise with age, attaining maximum values in the seventies, to decline thereafter? The variation in the concentration data precludes a definitive response, but further inquiry seems warranted. It is noteworthy that this trend of increasing concentration with age is present also in the data of Prout *et al.* (1959) for their nine patients with benign hyperplasia. Perhaps this rise in $^{65}$Zn with age is related to the corresponding increase in weight of the prostate and its decrease sometime during the eighth decade (Simmonds, 1918). In this connection, mention should be made of the finding of Fischer *et al.* (1955) that the stable zinc concentration of the dorsolateral prostate of the rat increases with age, reaching a maximum at about 160 days. Gunn *et al.* (1955) also found that the prostates of rats six months and older picked up the largest amounts of $^{65}$Zn.

TABLE VII.—*$^{65}$Zn Concentration (per cent dose/g. wet weight* $\times$ $10^{-3}$) *in the Prostates of* 44 *Patients as a Function of Age*

| Decade | Number of patients | Average | Range |
|---|---|---|---|
| 30–39 | 1 | 4·46 | — |
| 40–49 | 1 | 5·60 | — |
| 50–59 | 6 | 6·91 | 1·87–14·7 |
| 60–69 | 7 | 10·82 | 2·44–21·2 |
| 70–79 | 22 | 11·71 | 3·26–31·5 |
| 80–89 | 7 | 9·40 | 5·82–13·7 |

*Cellular and intracellular $^{65}$Zn in the prostate.*—The wide fluctuation in $^{65}$Zn content in the prostate gland, determined grossly by sample counting, is also found on a histological level by radioautography, which demonstrates that the follicles differ in their affinity for $^{65}$Zn. Histochemical confirmation is provided by the studies performed with dithizone staining of the prostates of developing rats (Fischer *et al.*, 1955). As the rats become able to breed (about 50 days), a few areas within isolated acini become stainable. Several days thereafter, scattered acini are seen in which all the cells react positively. Spotty activity of follicles has been encountered in the study of other glands, an outstanding example being the thyroid gland with respect to its variable localization of $^{131}$I.

Some of the other findings from our radioautographs are also in good agreement with the literature. The localization of $^{65}$Zn by the border epithelium is in accord with the radioautograph published by Daniel *et al.* (1956) for human prostate and with those presented by Wetterdal (1958) and Millar, Vincent and Mawson (1961) for the dorsolateral prostate of the rat. Using the dithizone stain, Mager *et al.* (1953) studied the prostates of the dog, man, monkey and rabbit, reporting that the apical portions of the secretory cells were most intensely stained. They found that the associated connective and muscle tissues did not contain zinc. Employing a dithizone complex-forming solution to enhance specificity for zinc, this group showed, also, that the nuclei were non-staining.

Fleischhauer (1957) has also observed stainable zinc in the apical border of prostatic cells in the rat, but the distal portions of the epithelium concentrated the metal only slightly ; the periphery of the nucleus was occasionally positive for zinc. Gunn and Gould (1956) published a photomicrograph which demonstrated positive staining of the acini at the luminal border in the lateral prostate of the rat. Rixon and Whitfield (1959) confirmed this high concentration of zinc in lateral portions of the dorsolateral prostate of the rat, finding three regions with positive staining : apical cytoplasm, nucleoli and sub-epithelial stroma.

In spite of the evidence just cited and confirmed by our radioautographs, the observation that the nuclei of the apical epithelium contain little or no $^{65}$Zn is unexpected, in view of the distribution of stable zinc in animal tumours and livers. Heath (1949) and Heath and Liquier-Milward (1950) have reported that zinc is associated with nucleoprotein from tumour tissues. Zinc has been found also in the nuclear fractions of normal rat livers (Thiers and Vallee, 1957) and of mouse mammary tumours and livers (Bartholomew, Tupper and Wormall, 1959). It should be remarked, however, that Rosenfeld and Tobias (1951) were unable to detect nucleoprotein-bound zinc in their studies.

The radioautographs indicate that both holocrine and apocrine types of follicles can actively pick up $^{65}$Zn. As already noted, Gunn and Gould (1957) have shown that the lateral tips of the dorsolateral prostate, which are concerned with the localization of the radioisotope and devoid of fructose, are comprised exclusively of apocrine type acini. Conversely, the dorsal tips, which have a higher fructose content and reduced $^{65}$Zn levels, have only holocrine units. The situation in man is, apparently, unlike that which prevails in the rat.

### SUMMARY

The distribution of $^{65}$Zn was studied in the organs of 14 patients at autopsy, occurring between 1 and 174 days following administration of 100 $\mu$c. The greatest concentration of the radioisotope is reached within a few days in the liver (about 0·050 per cent dose/g. wet weight), which localizes about 2–8 times more than the pancreas and as much as 10–30 times more than muscle. $^{65}$Zn leaves the liver slowly, 0·013 per cent dose/g. still being present 81 days after infusion. A more rapid turnover of the administered radioisotope occurs in the pancreas. In this organ, the maximum concentration (about 0·023 per cent dose/g.) is attained by the second day ; within a week, the level of the tagged metal has fallen by two-thirds, and by 81 days, hardly a tenth remains. The maximum concentration of $^{65}$Zn in the spleen (0·013 per cent dose/g.) is about half that of the pancreas. For the spleen, as well as for those organs having even lower levels of the radioisotope (seminal vesicles, lung, bladder and muscle), no temporal relationship could be discerned.

The prostatic localization of $^{65}$Zn was studied by assaying autopsy specimen from 8 of these patients and surgical specimens from 36 others undergoing surgery. For the entire series of 44 patients having normal, hyperplastic, or malignant prostates, the mean $^{65}$Zn concentration is 0·00992 $\pm$ 0·00615 per cent dose/g., ranging between 0·0315 and 0·00150 ; these $^{65}$Zn values are second only to those of the liver. No correlation could be demonstrated between the concentration of the radiometal and the condition of the prostate. By assaying samples of different lobes from the hyperplastic glands of 15 patients, marked variation in $^{65}$Zn concentrations is encountered within the organ unrelated to the anatomical subdivisions. Radioautographs of prostatic sections reveal that the deposition of the radioisotope, which is largely confined to the border epithelial cells, is not uniform. The smaller follicles accumulate the radioisotope predominantly, with both apocrine and holocrine type acini participating. Lesser amounts of the tagged metal are found in the lumens and ducts. $^{65}$Zn is concentrated principally by the cytoplasm of these border epithelial cells, insignificant amounts being taken up by the nuclei and by the associated connective and muscle tissues.

Radioautographs, prepared from bone marrow aspirates obtained about one week after $^{65}$Zn infusion, show that megakaryocytes and all early forms of the hematopoietic series are very heavily labeled. Mature leucocytes take up some $^{65}$Zn, but comparatively little of the nuclide is present in the erythrocytes.

The distribution pattern of the radioisotope is discussed in relation to stable zinc determinations in man. Our $^{65}$Zn data are compared also to those reported for experimental animals. Assuming uniform concentration throughout the organ, estimates are made of total organ uptake of $^{65}$Zn. The maximum accumulation of the administered tracer occurs in skeletal muscle and in liver, while pancreas and prostate have by far the lowest uptake. The effect of some factors on prostatic levels of $^{65}$Zn is considered. The intracellular deposition of $^{65}$Zn by the prostate of man is compared to the histochemical studies of stable zinc in man and experimental animals.

The authors wish to acknowledge the excellent technical assistance rendered by Messrs. Joseph V. Marino and Laszlo Grosz. This investigation was supported in part by Grant No. CY-4332 from the National Cancer Institute of the National Institutes of Health, U.S. Public Health Service.

### REFERENCES

Ballou, J. E.—(1959) 'Metabolism of $Zn^{65}$ in the Rat'. Richland, Washington (Hanford Atomic Products Operation), HW-60062.

Bartholomew, M. E., Tupper, R. and Wormall, A.—(1959) *Biochem. J.*, **71**, 15.

Boyd, G. A.—(1955) 'Autoradiography in Biology and Medicine'. New York (Academic Press), p. 248.

Daniel, O., Haddad, F., Prout, G. and Whitmore, W. F.—(1956) *Brit. J. Urol.*. **28**, 271.

Dennes, E., Tupper, R. and Wormall, A.—(1961) *Biochem. J.*, **78**, 578.

Fischer, M. I., Tikkala, A. O. and Mawson, C. A.—(1955) *Canad. J. Biochem. Physiol.*, **33**, 181.

Fleischhauer, K.—(1957) *Naturwissenschaften*, **44**, 589.

Fredricks, R. E., Tanaka, K. R. and Valentine, W. N.—(1960) *J. clin. Invest.*, **39**, 1651.

Gilbert, I. G. F. and Taylor, D. M.—(1956) *Biochim. biophys. Acta*, **21**, 545.

Graig, F. A. and Siegel, E.—(1960) *Proc. Soc. exp. Biol., N.Y.*, **104**, 391.

Gunn, S. A. and Gould, T. C.—(1956) *Ibid.*, **92**, 17.—(1957) *Anat. Rec.*, **128**, 41.

*Iidem*, Ginori, S. S. and Morse, J. G.—(1955) *Proc. Soc. exp. Biol., N.Y.*, **88**, 556.

Heath, J. C.—(1949) *Nature*, **164**, 1055.

*Idem* and Liquier-Milward, J.—(1950) *Biochim. biophys. Acta*, **5**, 404.

Herring, W. B., Leavell, B. S., Paixao, L. M. and Yoe, J. H.—(1960) *Amer. J. clin. Nutr.*, **8**, 855.

Hoare, R., Delory, G. E. and Penner, D. W.—(1956) *Cancer*, **9**, 721.

KERR, W. K., KERESTECI, A. C. AND MAYOH, H.—(1960) *Ibid.*, **13**, 550.
MCCANCE, R. A. AND WIDDOWSON, E. W.—(1942) *Biochem. J.*, **36**, 392.
MCISAAC, R. J.—(1955) *Endocrinology*, **57**, 571.
MAGER, M., MCNARY, W. F., Jr. AND LIONETTI, F.—(1953) *J. Histochem. Cytochem.*, **1**, 493.
MAWSON, C. A. AND FISCHER, M. I.—(1952) *Canad. J. med. Sci.*, **30**, 336.
MESCHAN, I., QUINN, J. L., WITCOFSKI, R. L. AND HOSICK, T. A.—(1959) *Radiology*, **73**, 62.
MILLAR, M. J., VINCENT, N. R. AND MAWSON, C. A.—(1961) *J. Histochem. Cytochem.*, **9**, 111.
NATIONAL BUREAU OF STANDARDS.—(1959) Handbook 69, 'Maximum Permissible Body Burdens and Maximum Permissible Concentrations of Radionuclides in Air and in Water for Occupational Exposure'. Washington (U.S. Government Printing Office).
PELC, S. R.—(1956) *Int. J. appl. Radioisotopes*, **1**, 172.
PROUT, G., Jr., SIERP, M. AND WHITMORE, W. F., Jr.—(1959) *J. Amer. med. Ass.*, **169**, 1703.
RIXON, R. H. AND WHITFIELD, J. F.—(1959) *J. Histochem. Cytochem.*, **7**, 262.
ROSENFELD, I. AND TOBIAS, C. A.—(1951) *J. biol. Chem.*, **191**, 339.
ROSS, J. F., EBAUGH, F. G., Jr. AND TALBOT, T. R., Jr.—(1958) *Trans. Ass. Amer. Physcns.*, **71**, 322.
SHELINE, G. E., CHAIKOFF, I. L., JONES, H. B. AND MONTGOMERY, M. L.—(1943) *J. biol. Chem.*, **149**, 139.
SIMMONDS, M.—(1918) *Frankfurt. Z. Path.*, **21**, 178.
THIERS, R. E. AND VALLEE, B. L.—(1957) *J. biol. Chem.*, **226**, 911.
TIPTON, I. H.—(1960) The Distribution of Trace Metals in the Human Body. *In* 'Metal-Binding in Medicine'. Ed. Seven, M. J. and Johnson, L. A. Philadelphia (Lippincott).
VALLEE, B. L.—(1955) Zinc and Metalloenzymes. *In* 'Advances in Protein Chemistry'. Ed. Anson, M. L., Bailey, V. and Edsall, J. T. New York (Academic Press).—(1959) *Physiol. Rev.*, **39**, 443.
*Idem* AND GIBSON, J. G.—(1948) *J. biol. Chem.*, **176**, 445.
VIKBLADH, I.—(1951) *Scand. J. clin. lab. Invest.*, Suppl., **2**, 9.
WETTERDAL, B.—(1958) *Acta Radiol.*, Suppl., **156**, 1.
WIDDOWSON, E. W., MCCANCE, R. A. AND SPRAY, C. M.—(1951) *Clin. Sci.*, **10**, 113.